# An astrocytic AMPAR-dependent parity signal preserves synaptic strength

**DOI:** 10.64898/2026.10.08.757679

**Published:** 2026-10-11

**Authors:** Nimrod Elazar, J. Russell Ravenel, Jinhu Kim, Trisha Vaidyanathan, Brenda C. Shields, Olivia Wirfel-Farenchak, Kunihiro Uryu, Shaun S.X. Lim, James M. Roach, Weizhan Zhang, Xiangyu Cao, Micah E. van Praag, Haidun Yan, Guoqiang Yu, Michael R. Tadross, Cagla Eroglu

**Affiliations:** 1 Department of Cell Biology, Duke University School of Medicine; Durham, NC, USA.; 2 Howard Hughes Medical Institute, Chevy Chase, MD, USA.; 3 Aligning Science Across Parkinson’s (ASAP) Collaborative Research Network; 4 Department of Neurobiology, Duke University School of Medicine; Durham, NC, USA.; 5 Department of Biomedical Engineering, Duke University Pratt School of Engineering; Durham, NC, USA.; 6 Department of Automation, Tsinghua University, Beijing 100084, China.; 7 IDG/McGovern Institute for Brain Research, Tsinghua University, Beijing 100084, China.; 8 Department of Neurosurgery, Duke University School of Medicine; Durham, NC, USA.; 9 HHMI Janelia Research Campus, Ashburn, VA, USA.

## Abstract

Astrocytic processes closely associate with excitatory synapses and express AMPARs, positioning them to sense the same glutamate signals as neurons. Whether a mismatch between neuronal and astrocytic glutamate sensing affects synaptic physiology is unknown. Using AstroHTP, an extension of Drug Acutely Restricted by Tethering (DART) for cell-type-specific pharmacology, we found that acute astrocytic AMPAR inhibition spared neuronal AMPARs and basal transmission yet converted ongoing glutamatergic activity into a remarkably potent form of long-term depression (LTD) in mouse striatal projection neurons (SPNs). Unlike canonical presynaptic LTD, this form of LTD manifests postsynaptically, requires non-ionotropic NMDA receptor signaling, and arises when glutamate transmission occurs without a matching astrocytic signal. Astrocytic glutamate sensing through AMPARs, therefore, acts as a parity signal that verifies transmission and stabilizes active synapses.

In the striatum, afferents from cortex and thalamus provide excitatory glutamatergic input to spiny projection neurons (SPNs), which sense glutamate rapidly through AMPA-type glutamate receptors (AMPARs) (1–3). Astrocytic processes closely associate with these synapses (4–7) and also express AMPARs, positioning them to sense the same rapid glutamate signals. Yet whether a mismatch between neuronal and astrocytic glutamate sensing affects synaptic physiology remains unknown.

Previous work shows that astrocytic AMPARs are functional and capable of detecting physiologically relevant glutamatergic activity (8–10). Functional Ca^2+^-permeable AMPARs have also been identified on astrocytes, where they mediate intracellular calcium signaling (9). However, causal studies have been difficult to implement. Inducible deletion of GluA1 and GluA4 from cerebellar Bergmann glia alters synaptic coverage, Purkinje cell currents, and motor coordination (10). Yet because receptor loss unfolds over weeks, glial remodeling and compensatory changes in gene expression complicate a direct causal link to astrocytic AMPARs themselves. Conversely, pharmacology is acute but cannot isolate the function of astrocytic AMPARs from their more abundant neuronal counterparts.

To overcome these limitations, we adapted Drug Acutely Restricted by Tethering (DART), a cell-specific pharmacology platform (11, 12), to acutely antagonize endogenous AMPARs in striatal astrocytes. Using this approach, we found that acute astrocytic AMPAR inhibition spared neuronal AMPAR function and basal transmission but converted ongoing glutamatergic activity into a noncanonical postsynaptically expressed form of long-term depression (LTD). Thus, astrocytic AMPARs sense synaptic glutamate and determine whether active synapses retain their strength or undergo depression. We propose that astrocytic glutamate sensing acts as a parity signal that verifies the fidelity of synaptic transmission.

## Establishing astrocytic DART for cell-type-specific pharmacology in intact circuits.

DART enables cell-specific pharmacology through two components: a cell-surface HaloTag Protein (HTP) expressed on cells of interest, and a drug linked by polyethylene glycol (PEG) to the HaloTag Ligand (HTL) (Fig. 1A). Covalent binding between HTP and HTL locally accumulates the tethered drug to concentrations substantially higher than ambient levels. The second-generation DART.2 platform achieves thousandfold enrichment of tethered drugs within minutes, enabling potent modulation of endogenous receptors in defined neuron types *in vivo* (11, 12). However, DART has not yet been extended to astrocytes.

We therefore developed AstroHTP, an AAV construct that achieves astrocyte–specific expression without the need for transgenic animals or recombinases. AstroHTP uses the astrocyte-specific GfaABC1D promoter (13) to express GPI-anchored HTP (HTP_GPI_) along with a bicistronic nuclear fluorescent reporter (IRES-^NLS^dTomato). To further enhance astrocyte specificity, we incorporated four repeats of a microRNA targeting cassette (4×6T), previously shown to suppress expression in neurons and endothelial cells (14) (Fig. 1B), and packaged the vector into the astrocyte-tropic PHP.AX capsid (15).

To assess expression penetrance and astrocyte specificity, we injected AstroHTP into the dorsal striatum (dSTR) of 8-week-old C57BL/6J mice and analyzed tissue 3 weeks later. We quantified viral expression using ^NLS^dTomato and determined cell-type identity by co-staining with nuclear markers of astrocytes (SOX9) (16), neurons (NeuN), and oligodendrocyte-lineage cells (OLIG2). AstroHTP labeled 53.2% ± 8.9% of dorsal striatal astrocytes (Fig. 1C–D). We did not detect dTomato expression in NeuN or OLIG2-positive nuclei. Thus, AstroHTP drove efficient, highly astrocyte-specific expression of HTP in striatal astrocytes.

We next asked whether HTP-expressing astrocytes effectively capture DART ligands *in vivo*. We infused the HTL-linked tracer dye, Alexa647^DART^, into the dSTR via an indwelling cannula and performed histology 24 hours later. Alexa647^DART^ accumulated robustly on ^NLS^dTomato-positive astrocytes, indicating efficient target engagement *in vivo* (Fig. 1E).

We next asked how long AstroHTP persists at the cell surface by measuring its turnover *in vivo* using a pulse-chase strategy. Biotin^DART^ was infused into the dSTR, followed by Alexa647^DART^ at intervals ranging from 1 to 72 hours. The remaining surface Biotin^DART^ was detected with streptavidin-Alexa750 (SA750) (Fig. 1F) and plotted as a function of chase interval (Fig. 1G). The decay was best fit by a double exponential (effective half-life: 1.41 ± 0.43 days), with a minor fast component accounting for the remaining 16% (half-life: 0.03 days) and a slow component accounting for 84% of the signal (half-life: 1.88 days). Thus, in astrocytes, most surface HTP persists for days, with a smaller, rapidly internalizing pool.

To confirm that astrocytes capture DART ligands onto Perisynaptic Astrocyte Processes (PAPs) at high resolution, we used a sparse transduction strategy, co-injecting a low-titer astrocyte-specific Cre virus with a high-titer Cre-dependent HTP-GPI-IRES-dTomato-farnesyl construct, in which farnesylated dTomato labels the inner membrane leaflet to enable reconstruction of the astrocyte's surface. Following striatal Alexa647^DART^ infusion, the tracer signal colocalized robustly with dTomato-labeled astrocytic membranes (Fig. 1H), confirming ligand capture and establishing DART labeling as a proxy for the ramified architecture of astrocytic processes.

To further confirm the localization of AstroHTP to PAPs at ultrastructural resolution, we performed immuno-electron microscopy (immuno-EM). HTP-expressing striatal astrocytes were labeled *in vivo* with Biotin^DART^ (Fig.1I). Brains were fixed four hours later, and sections were incubated with Alexa Fluor 647-FluoroNanogold^™^-streptavidin to label Biotin^DART^-positive astrocytes. Regions of interest containing Alexa647 fluorescence were selected and prepared for EM (Fig. 1J, fig. S1A). EM revealed that nanogold particles were concentrated in PAPs and excluded from neuronal pre- and postsynaptic compartments (Fig. 1K, fig. S1B). Some of the AstroHTP-bound nanogold particles were detected intracellularly, consistent with internalization.

Finally, we tested whether AstroHTP could support DART ligand capture in other brain regions. AstroHTP was injected into the hippocampus or motor cortex, followed three weeks later by Alexa647^DART^ infusion. In both regions, Alexa647^DART^ robustly accumulated on ^NLS^dTomato-positive astrocytes, with no apparent labeling in neurons or oligodendrocyte-lineage cells (fig. S1C-E). Together, these results establish AstroHTP as an effective, astrocyte-specific DART platform that can be generalized across brain regions.

## Astrocyte-specific inhibition of AMPAR activity using YM90K^DART^.

Transcriptomic and proteomic profiling studies show that striatal astrocytes express AMPAR subunits (17, 18) (Fig. S2A–B). Yet the functions of astrocytic AMPARs have remained difficult to test directly. AstroHTP provides a direct way to interrogate astrocytic AMPARs when paired with YM90K.1^DART.2^ (hereafter, YM90K^DART^), a tetherable competitive AMPAR antagonist (11, 12). Previous studies in HTP-positive neurons showed that low-dose exposure achieves potent AMPAR inhibition, because the concentration needed to drive drug tethering is approximately 300-fold lower than the reagent’s 50% ambient concentration (*AC*_50_) of 6 μM (fig. S2C). Once tethered, YM90K^DART^ is highly selective for AMPARs, with minimal off-target activity across other glutamate and non-glutamate receptors (12).

We tested whether YM90K^DART^ inhibits astrocytic AMPAR function in acute slices by using astrocytic calcium imaging as the readout (8, 9). 8-week-old C57BL/6J mice were injected with AstroHTP together with GfaABC1D-GCaMP6f, a fluorescent sensor of the cytosolic calcium concentration (19). AMPA (100 μM) was bath applied together with a positive allosteric modulator, cyclothiazide (CTZ, 100 μM), in the presence of tetrodotoxin (TTX, 0.5 μM) to inhibit neuronal action potentials. AMPA treatment under these conditions elicited robust astrocytic Ca^2+^ transients in striatal astrocytes, similar to those previously observed in cortical astrocytes (20). These responses were abolished by preincubation of slices with the AMPAR antagonist NBQX (30 μM), confirming that they required AMPAR activation (fig. S2D-F, Supp Video1–2). To determine if YM90K^DART^ can block AMPA-elicited astrocytic Ca^2+^ transients, slices were randomly assigned to preincubation with either 1 μM YM90K^DART^ or 1 μM Blank^DART^, a pharmacologically inert reagent (Fig. 2A-D). Both conditions also included 0.1 μM Alexa647^DART^, a fluorescent tracer that verifies ligand capture. Excess untethered ligand was washed out before imaging, ensuring that any effects of YM90K^DART^ reflect tethered, rather than ambient compound. Pre-incubation with YM90K^DART^ markedly reduced both the peak amplitude and the integrated Ca^2+^ signal in astrocytes relative to Blank^DART^ controls, confirming effective AMPAR antagonism (Fig. 2 E-G, fig. S2G, Supp Video3–4). We next examined the impact of YM90K^DART^-mediated astrocytic AMPAR inhibition on spontaneous astrocytic Ca^2+^ transients *ex vivo*. Striatal slices were again pre-treated with either YM90K^DART^ or Blank^DART^ but imaged in the absence of TTX, AMPA, or CTZ. Ca^2+^ events were analyzed with Activity Quantification and Analysis 2 (AQuA2), a machine-learning–based pipeline for resolving complex spatiotemporal calcium signals (21). YM90K^DART^ significantly reduced the frequency of spontaneous astrocytic Ca^2+^ events compared to slices treated with Blank^DART^, while mean event amplitude was unchanged (Fig. 2H-J, Supp Video 5–6). Thus, AstroHTP combined with YM90K^DART^ selectively inhibits astrocytic AMPAR signaling in striatal circuits.

## Unilateral inhibition of astrocytic AMPARs induces an ipsiversive turning bias.

To determine whether astrocytic AMPAR signaling contributes to striatal-dependent behavior *in vivo*, we asked whether attenuating astrocytic AMPAR signaling alters locomotion in the open field. To maximize behavioral sensitivity, we focused on a unilateral perturbation with turning bias as the primary behavioral outcome. We injected AstroHTP into the left dSTR and implanted a cannula at the same location for intracranial ligand infusion. Three weeks after surgery, mice received direct striatal infusions of either 30 μM YM90K^DART^ or a control cocktail containing 30 μM untetherable YM90K^PEG36^ and 30 μM Blank^DART^. In both conditions, 3 μM Alexa647^DART^ was included to assess target engagement. Critically, because both conditions have the same quantity of PEG-linked YM90K, any ambient effects are matched across conditions. The HTP tethering process is likewise matched because equivalent amounts of PEG-linked HTL were used, leaving the presence or absence of a physical linkage between YM90K and HTL as the key experimental variable (Fig. 3A). To assess motor output, we recorded freely moving mice from both groups in an open-field arena for two 30-minute sessions, at 1 hour and 24 hours post-infusion, tracking 18 body parts using 3-D Aligned Neural Network for Computational Ethology (DANNCE) (22, 23) (Supp Video 7). YM90K^DART^-treated mice developed a directional bias 1 h post-infusion in the first session. This bias reflects two overlapping effects: cannula implantation and virus expression caused a contraversive (rightward) turning bias in controls (Fig. 3B), and YM90K^DART^ counteracted this bias by promoting turning in the ipsiversive (leftward) direction (Fig. 3B-D). This shift occurred despite no change in total turns (Fig. 3E) or total distance traveled (Fig.3F). By 24 hours post-infusion, the turning bias had largely resolved, with a trending but nonsignificant difference between groups (fig. S3A-D), suggesting that compensatory mechanisms may rebalance the circuit over time, underscoring the importance of an acute perturbation. Other secondary metrics, including distance traveled, time spent in the center, and time spent immobile, showed no effect of YM90K^DART^ relative to controls in either open-field session (fig. S3E-G). Given that contraversive movements require activity across direct- and indirect-pathway spiny projection neurons (24), the observed reduction in contraversive turns induced by astrocytic YM90K^DART^ suggests a net reduction in SPN activity.

## Astrocyte-tethered YM90K^DART^ does not act transcellularly on neuronal AMPARs.

We next asked whether an astrocyte-tethered YM90K^DART^ could act transcellularly to inhibit neuronal AMPARs. Drd1a-Cre or Adora2a-Cre mice were injected with AAV-hSyn-DIO-GFP to label genetically identified direct- or indirect-pathway SPNs (dSPN or iSPN, respectively), while striatal astrocytes were transduced with AstroHTP. Three to four weeks later, we prepared acute coronal brain slices and obtained whole-cell voltage-clamp recordings from GFP-positive SPNs adjacent to ^NLS^dTomato^+^ astrocytes (Fig. 3G-H). We isolated neuronal AMPAR-mediated currents by pharmacologically blocking GABA_A_ and NMDA receptors. Evoked EPSCs (eEPSCs) were elicited every 20 seconds to monitor AMPA-mediated synaptic responses. After a stable 5-minute baseline, we bath-applied YM90K^DART^ or the control cocktail for 15 minutes, followed by a 10-minute washout during which recordings continued (Fig. 3I). Under these conditions, YM90K^DART^ did not alter eEPSC amplitude in either dSPNs (Fig. 3J-L) or iSPNs (Fig. 3M-O). By contrast, when HTP was expressed in SPNs, the neuronally-tethered YM90K^DART^ robustly reduced AMPAR-mediated eEPSC amplitude (fig. S4). Thus, astrocyte-tethered YM90K^DART^ does not appreciably cross the perisynaptic cleft to engage neuronal AMPARs, consistent with previously shown membrane-confined action (11, 12, 25).

## Astrocytic glutamate sensing through AMPARs is necessary to maintain synaptic strength.

We next repeated the experiment in the absence of NMDAR antagonism. Under these conditions, inhibition of astrocytic AMPARs via YM90K^DART^ produced a robust reduction in eEPSC amplitude in both dSPNs (Fig. 3P-R) and iSPNs (Fig. 3S-U). This suppression persisted after washout of free ligand, further confirming that the effect arises from stable tethering of YM90K^DART^ to AstroHTP rather than from residual ambient compound. These results suggest that astrocytic AMPAR inhibition induces a robust, NMDAR-dependent decrease in synaptic strength that resembles long-term depression (LTD) (26).

To determine whether this decrease in synaptic strength requires ongoing synaptic transmission (27, 28), we compared continuous afferent stimulation throughout YM90K^DART^ application to a protocol in which afferent stimulation was paused during YM90K^DART^ application and resumed following washout (Fig. 4A). As before, YM90K^DART^ treatment reduced eEPSC amplitude when paired with ongoing synaptic stimulation (Fig. 4B-D). By contrast, when stimulation was paused during YM90K^DART^ application, the first post-drug eEPSC was unchanged from baseline (Fig. 4B-C, E-F), and eEPSC depression reemerged following the resumption of stimulation (Fig. 4G-H, fig. S6A). Similarly, astrocytic AMPAR inhibition had no effect on the amplitude or frequency of miniature excitatory postsynaptic currents (mEPSCs) in the absence of NMDAR antagonism without evoked stimulation (fig. S5). Taken together, these results show that blocking astrocytic AMPARs reduces synaptic strength in an activity-dependent manner, consistent with a use-dependent LTD-like phenomenon.

In the striatum, LTD is canonically expressed presynaptically, via a decrease in glutamate release probability (29), and a resulting increase in the paired pulse ratio (PPR; second divided by first eEPSC amplitude). We thus repeated the assay in dSPNs adjacent to AstroHTP-expressing astrocytes while measuring PPRs (Fig. 4I-J). As before, astrocytic YM90K^DART^ induced a marked reduction in eEPSC amplitude (Fig. 4K, fig. S6B). Surprisingly, in contrast to canonical LTD in these cells, the PPR remained similar between control and YM90K^DART^-treated groups (Fig. 4L, fig. S6C). We then examined postsynaptic glutamate receptor function (Fig. 4M). We found that astrocytic AMPAR inhibition, combined with evoked stimulation in the absence of NMDAR blockers, significantly reduced the AMPAR/NMDAR ratio (Fig. 4N-O). Taken together, these findings indicate that the observed LTD is primarily driven by a reduction in postsynaptic AMPAR-mediated currents.

## Astrocyte-tethered YM90K^DART^ induces LTD via non-ionotropic NMDAR signaling

Although postsynaptic LTD is not typical for SPNs, it is the canonical form of LTD for pyramidal neurons, wherein NMDAR-dependent LTD classically requires Ca^2+^ influx, activating phosphatases that drive AMPAR internalization and a resulting drop in the AMPAR/NMDAR ratio (30, 31). However, NMDARs can also induce LTD non-ionotropically, wherein glutamate induces a conformational change that triggers intracellular signaling independent of ion flux (32).

Even though Mg^2+^ blocks ionotropic NMDAR signaling at our −70 mV holding potential (33), ionotropic signaling may still occur in spines or in dendritic compartments that are not fully space-clamped (34). Thus, to decisively assess whether ionotropic NMDAR signaling is required, we co-applied the NMDAR pore blocker MK-801 with YM90K^DART^ (Fig. 5A). MK-801 did not prevent the reduction in eEPSC amplitude induced by YM90K^DART^ (Fig. 5B-D, fig. S6D-E), indicating that ionotropic NMDAR signaling is not required for this form of LTD.

Non-ionotropic NMDAR signaling (35, 36) is strongly attenuated by NMDAR co-agonists (37), and D-serine is the main endogenous NMDAR co-agonist in the striatum (38, 39). We thus combined MK-801 with a saturating concentration of D-serine (10 μM) to suppress non-ionotropic NMDAR signaling. Under these conditions, astrocyte-tethered YM90K^DART^ no longer induced LTD (Fig. 5B-D, fig. S6F). These results indicate that astrocytic AMPAR inhibition mediates LTD via non-ionotropic NMDAR signaling and suggest that NMDAR co-agonists, such as D-Serine, are critical for maintaining synaptic strength during ongoing synaptic activity.

Finally, we tested whether NMDAR co-agonist depletion is sufficient to phenocopy astrocytic AMPAR inhibition. To do so, we bath-applied D-amino acid oxidase (40) (DAAO; 0.3 U/mL), an enzyme that degrades D-serine, glycine, and other NMDAR co-agonists, whilst using the same eEPSC recording paradigm in Fig. 3G. Importantly, we did not use YM90K^DART^ in these experiments. Under these conditions, DAAO alone reduced eEPSC amplitude to a similar extent as astrocytic YM90K^DART^ (Fig. 5E-H, fig. S6G-H), indicating that reduced NMDAR co-agonist availability is sufficient to recapitulate the effect of astrocytic YM90K^DART^. Together, these results show that an extracellular NMDAR co-agonist, such as D-serine, is required for preserving synaptic strength during ongoing synaptic activity. Taken together with previous results, these findings also suggest that astrocytic AMPARs maintain synaptic strength by regulating extracellular NMDAR co-agonist availability.

## Discussion:

A growing body of work positions astrocytes as an instructive element that can shape circuit formation and function in the striatum (40–43). However, the functional consequences of neurotransmitter sensing by perisynaptic astrocytic receptors remain poorly understood. Our results reveal a fundamental role for AMPAR-mediated astrocytic glutamate sensing as a parity signal required to preserve synaptic strength during use. In the absence of this parity signal, non-ionotropic NMDAR signaling induces a parity-mismatch form of LTD.

In the striatum, learning-related LTD is canonically instructed by spike timing and by dopamine, endocannabinoid, adenosine, and cholinergic signaling, and is expressed presynaptically (44–52). Parity-mismatch LTD is fundamentally different: it arises when glutamate transmission occurs without a matching astrocyte-dependent signal, and it is expressed postsynaptically. Whether it also contributes to learning-related plasticity remains unknown.

Our findings carry implications for striatal pathophysiology. Maladaptive changes in corticostriatal synaptic strength have been implicated in Parkinson's disease, levodopa-induced dyskinesia, and obsessive-compulsive disorder, which have all been associated with astrocytic involvement (18, 53–55). Loss of the astrocytic AMPAR-dependent parity signal induces LTD onto both direct- and indirect-pathway SPNs, suggesting a mechanism by which astrocytic dysfunction could globally suppress striatal gain (29, 50). Consistent with this possibility, striatal D-serine dysregulation has been documented in Parkinson's disease and levodopa-induced dyskinesia (56). More broadly, the astrocytic parity signal provides a framework whereby synaptic pathologies can arise from failed astrocytic function rather than a primary neuronal defect, a hypothesis that is now directly testable in disease models using AstroHTP.

Several mechanistic questions remain. Astrocytic AMPAR inhibition reduced the frequency of spontaneous astrocytic calcium events, but whether these dynamics contribute to parity-mismatch LTD is unresolved (57–59). It is also unclear if astrocytes regulate AMPAR trafficking within peripheral processes, analogous to neuronal activity-dependent trafficking (60 , 61), and whether astrocytic AMPAR inhibition alters the astrocytic membrane potential in ways that contribute to parity-mismatch LTD (62). The identity of the parity signal also remains open: D-serine is thought to predominate at striatal synapses, but DAAO can also oxidize other NMDAR coagonists such as glycine, albeit with a Km roughly 1000-fold above physiological glycine concentrations (63), so our enzymatic approach cannot fully distinguish the contributions of D-serine, glycine, or other D-amino acids to preserving synaptic strength downstream of astrocytic AMPAR engagement. Finally, why astrocytes, rather than neurons, provide the parity signal is a major question. Because each astrocyte ensheathes many synapses (64, 65), an astrocyte-derived parity signal may operate at a spatial scale larger than that of an individual synapse, positioning astrocytes to integrate local circuit context into parity signaling decisions. Defining the spatiotemporal spread of this signal remains an important goal of future work.

In conclusion, these findings identify an astrocytic parity signal that stabilizes synaptic strength by linking glutamate sensing to suppression of non-ionotropic NMDAR signaling. Astrocyte-targeted DART was essential for revealing this mechanism because it enables acute, cell-specific inhibition of endogenous receptors in intact circuits. Extending this strategy across other astrocytic receptor classes and disease models should reveal how these glial cells decode neurotransmitter signals to shape neural computation.

## Supplementary Material

Supplement 1

Supplement 2

## Figures and Tables

**Figure 1.**
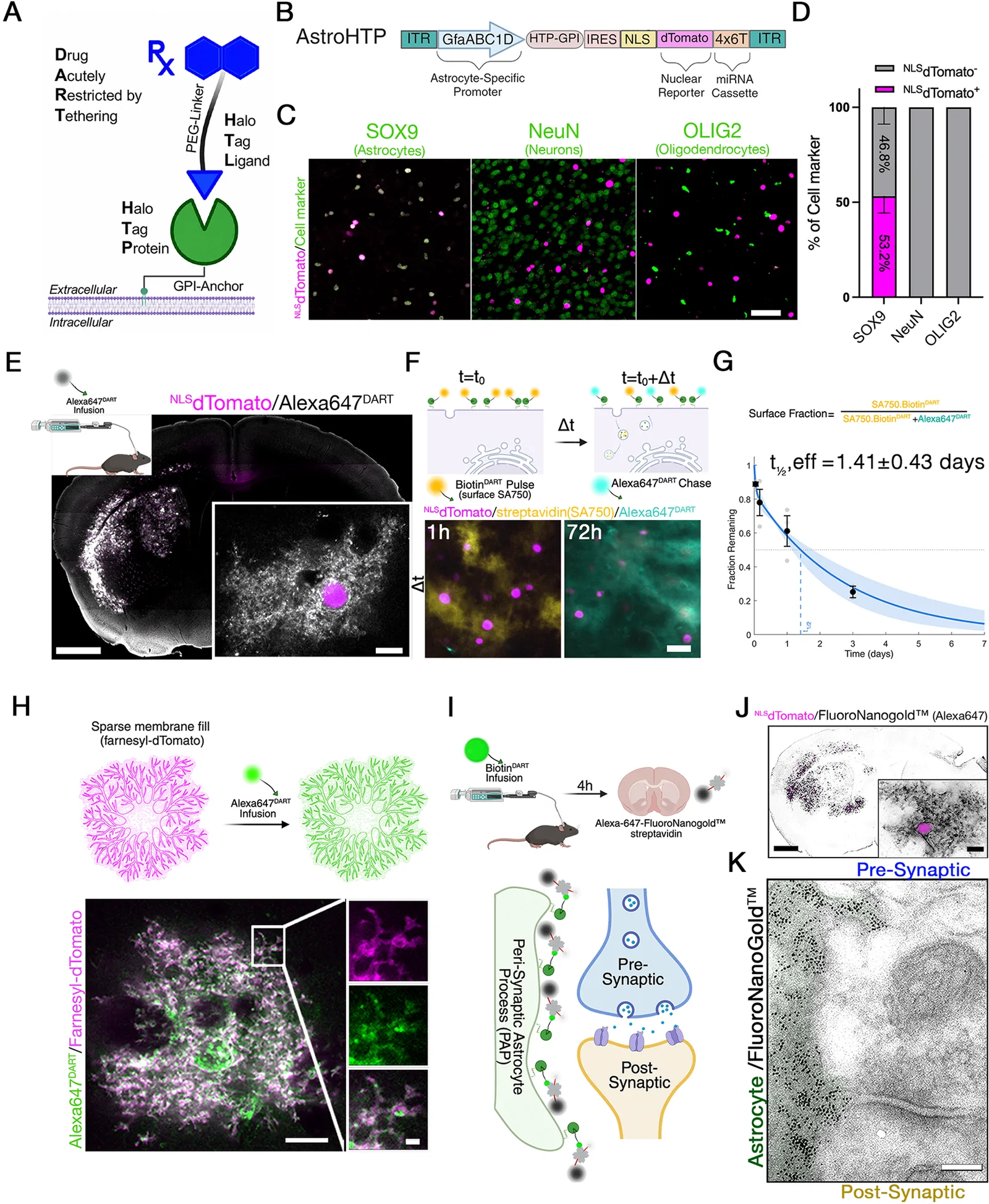
AstroHTP drives astrocyte-specific HaloTag expression and perisynaptic tethered-ligand capture in striatum **(A)** Schematic of DART technology. Genetically specified cells express a GPI-anchored HaloTag Protein (HTP), which acutely tethers a drug (Rx) attached via a polyethylene glycol linker (PEG) to a HaloTag Ligand (HTL). **(B)** Schematic of the AstroHTP viral construct. HTP-GPI is expressed under control of the GfaABC1D promoter, followed by an IRES sequence and a dTomato reporter containing a nuclear localization signal (NLS). A 4×6T microRNA-targeting cassette is added to the 3' UTR, and the construct is packaged in the PHP.AX capsid. **(C)** Three weeks post-AstroHTP injection, coronal mouse brain sections containing the dSTR were immunolabeled with antibodies against SOX9 (astrocytes), NeuN (neurons), and OLIG2 (oligodendrocytes). Scale bar, 100 μm. **(D)** Quantification of AstroHTP cell-type transduction efficiency, measured as the percentage of each cell type co-localized with dTomato-positive nuclei in the dSTR (N = 3 mice and 5 to 6 images per mouse). Data are mean ± SEM. **(E)** AstroHTP was injected into the dSTR of adult C57BL/6J mice. Three weeks later, 3 μM Alexa647 fluorophore conjugated to HTL (Alexa647^DART^) was infused into the striatum and histology was performed 24 h later. Representative coronal section, showing ligand capture (grey) and NLS-dTomato (magenta). Scale bars: 500 μm, 5 μm. **(F-G)** HTP turnover, **(F)** Experimental pulse-chase approach: a 0.9 μL pulse of 60 μM biotin-conjugated HTL (Biotin^DART^) was administered, followed 1–72 h later by a 0.9 μL chase of 60 μM Alexa647^DART^. Example histology at the 1 h and 72 h chase intervals. Scale bar, 20 μm. **(G)** Quantification of the fraction of original surface HTP signal remaining versus chase interval; each symbol represents an independent mouse, and error bars are mean ± SEM. Data were fit with a double exponential, yielding an effective half-turnover time for HTP of 1.41 ± 0.43 days (mean ± 95% CI, intervals derived from the 2.5th and 97.5th percentiles of the fitted parameter distribution). **(H)** Astrocytes capture DART ligands on their fine processes. A Cre-dependent HTP-GPI variant with a membrane-targeted reporter (dTomato-farnesylated) was co-injected with GfaABC1D-Cre into the dSTR of adult C57BL/6J mice. Three weeks later, 3 μM Alexa647^DART^ was infused into the dSTR of head-fixed mice, and histology was performed 24 h later. Representative coronal section showing DART ligand capture (green) and membrane-targeted dTomato (magenta). Scale bars: 10 μm; inset, 1 μm. **(I-K)** AstroHTP-transduced astrocytes capture DART ligands at perisynaptic processes. **(I)** AstroHTP was injected into the dSTR of adult C57BL/6J mice. Three weeks post-injection, 0.9 μL of 30 μM Biotin^DART^ was infused into the striatum, and histology was performed 4 h later. Coronal sections were processed for immuno-electron microscopy and labeled with Alexa647-conjugated FluoroNanogold^™^. Schematic of the tripartite synapse depicting perisynaptic Alexa647-conjugated FluoroNanogold^™^ capture **(J)** Representative coronal section showing immunohistochemistry for NLS-dTomato (magenta) and FluoroNanogold (black). Scale bars: 1000 μm, 10 μm. **(K)** A representative electron micrograph of the mouse striatum showing a nanogold-labeled perisynaptic astrocytic process (green). Scale bar, 100 nm.

**Figure 2.**
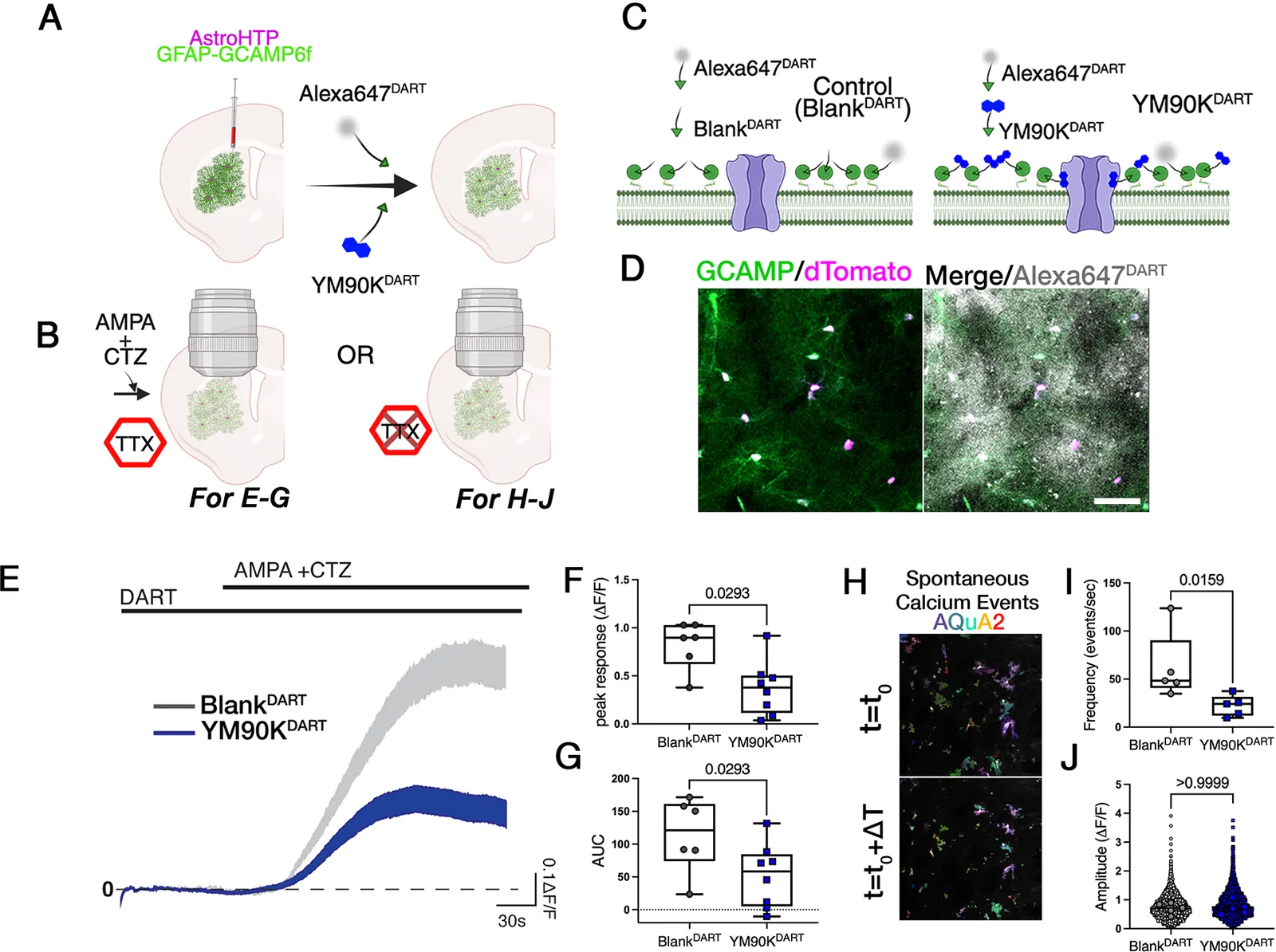
Inhibition of astrocytic AMPARs via YM90K^DART^ **(A)** Experimental schematic for inhibiting astrocyte AMPARs. Coronal striatal sections co-expressing AstroHTP and GFAP-GCaMP6f were pre-incubated with DART ligands. **(B)** Experimental schematic comparing two conditions: AMPA-evoked Ca^2+^ responses (100 μM AMPA + 100 μM cyclothiazide [CTZ] + 0.5 μM TTX; panels E–G) versus spontaneous Ca^2+^ signaling (AMPA omitted, TTX excluded; panels H–J). **(C)** Sections were incubated with either 1 μM Blank ^DART^ or 1 μM YM90K^DART^, together with 0.1 μM Alexa647^DART^ to visualize drug capture**. (D)** Representative image showing AstroHTP (dTomato, magenta) co-expressed with GCaMP6f (green) and Alexa647^DART^ capture (grey). Scale bar, 50 μm. **(E-G)** YM90K^DART^ attenuates AMPA-evoked Ca^2+^ responses in AstroHTP astrocytes. **(E)** ΔF/F traces showing Ca^2+^ responses of striatal astrocytes to bath application of 100 μM AMPA in the presence of 100 μM CTZ and 0.5 μM TTX. Traces correspond to slices pre-incubated with 1 μM Blank^DART^ (control, grey) or 1 μM YM90K^DART^ (blue). Shading represents mean ± SEM. **(F**) Summary of peak AMPA-evoked Ca^2+^ responses (ΔF/F): Blank^DART^, 0.822 ± 0.101; YM90K^DART^, 0.373 ± 0.0998 (Mann–Whitney test, P = 0.0293; n = 6 slices from 4 mice (Blank^DART^) and 8 slices from 5 mice (YM90KDART); 96 and 127 ROIs, respectively**). (G)** Summary of AMPA-evoked Ca^2+^ response AUC: Blank^DART^, 114 ± 22.9; YM90K^DART^, 51.8 ± 17.1 (Mann–Whitney test, P = 0.0293; n = 6 slices from 4 mice (Blank^DART^) and 8 slices from 5 mice (YM90K^DART^); 90 and 127 ROIs, respectively). Peak and AUC were analyzed using the same rank-based test; because these measures are highly correlated across slices, they yield identical U-statistics and P-values. **(H–J)** Astrocytic AMPARs regulate spontaneous Ca^2+^ signaling. **(H)** Representative images of astrocytic Ca^2+^ events detected using AQuA2. **(I)** The frequency of astrocytic Ca^2+^ events was reduced following YM90K^DART^ treatment (Blank^DART^ 62 ± 16; YM90K^DART^ 22 ± 4.8 events/s; Mann–Whitney test, P = 0.0159; n = 5 slices from 4 mice per condition). **(J)** Amplitude of astrocytic Ca^2+^ events was unchanged following YM90K^DART^ treatment (control 0.703 ± 0.0846; YM90K^DART^ 0.715 ± 0.104; Mann–Whitney test, P > 0.9999; n = 5 slices from 4 mice per condition).

**Figure 3.**
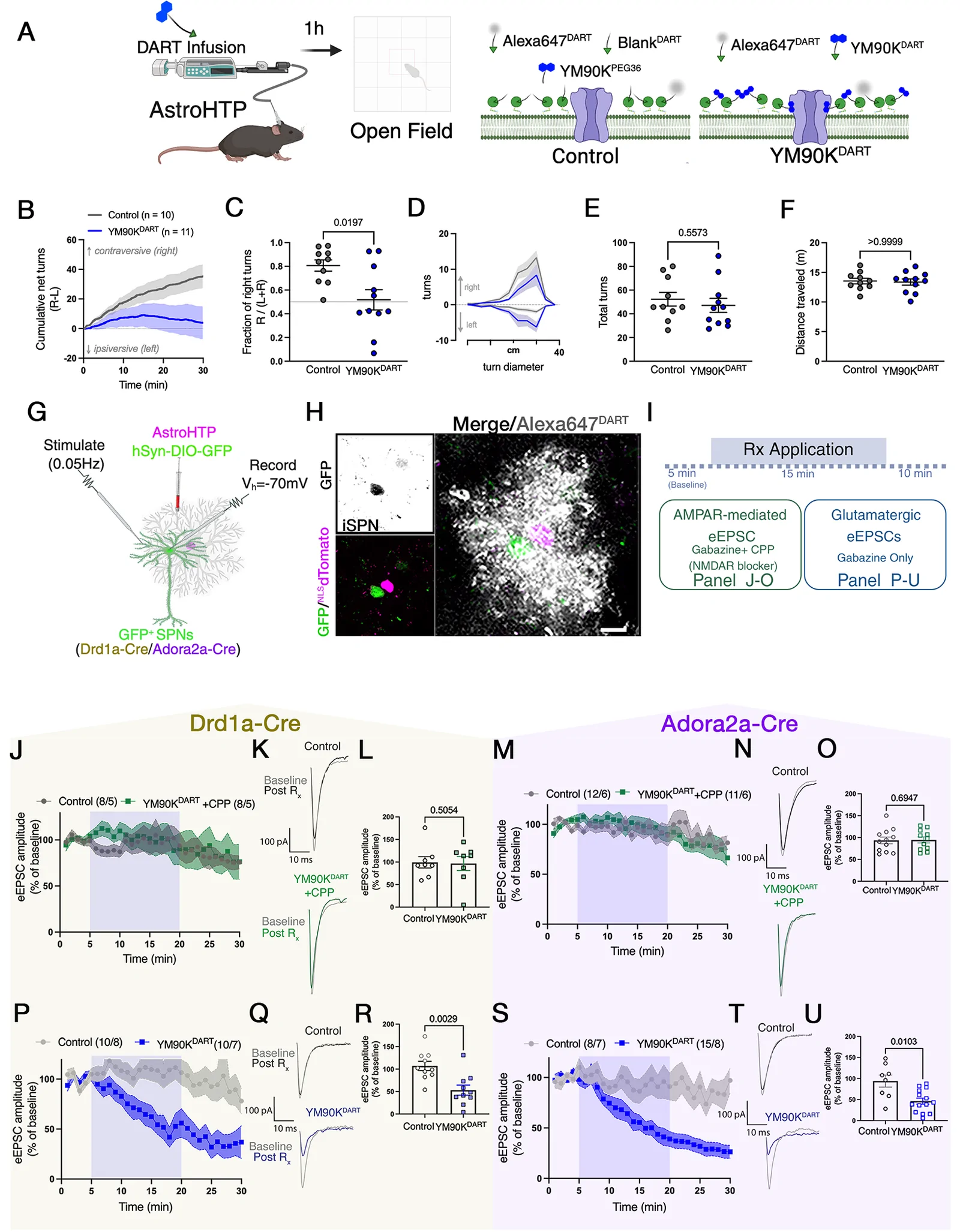
Astrocyte-specific inhibition of AMPARs reduces excitatory synaptic activity of SPNs in an activity-dependent and NMDAR-dependent manner. **(A)** Astrocytic AMPAR inhibition *in vivo*. Experimental schematic: adult C57BL/6J mice were injected with AstroHTP in the left dSTR and implanted with a cannula for intracranial DART infusion. Three weeks later, DART ligands were infused into head-fixed mice for 15 min, followed by open-field behavioral analysis. Mice were infused with 30 μM Blank^DART^ and 30 μM YM90K^PEG36^, or 30 μM YM90K^DART^, each together with 3 μM Alexa647^DART^. **(B-F)** Astrocytic AMPAR inhibition corrects cannula implantation contraversive (right) turning bias. **(B)** cumulative net turns of the mouse, showing both right and left turns over time and their accumulation over the session. Data are mean ± SEM, N = 10 control, 11 YM90K^DART^. **(C)** Fraction of right turns, treatment (Control 0.807± 0.0474; YM90K^DART^ 0.518± 0.0852; Mann–Whitney test, P = 0.0197; N = 10 control, 11 YM90K^DART^). **(D)** Turn-diameter histogram (1 cm bins) 1 h after control (grey) or YM90K^DART^ infusion. Left turns are plotted as downward bars and right turns as upward bars. Data are mean ± SEM, N = 10 control, 11 YM90K^DART^. **(E)** Total turns did not differ between groups (Control 52.3± 5.77; YM90K^DART^ 47.2± 5.92; Mann–Whitney test, P = 0.5573; N = 10 control, 11 YM90K^DART^). **(F)** Distance traveled did not differ between groups (Control 13.5± 0.475; YM90K^DART^ 13.4± 0.530 ; Mann–Whitney test, P >0.9999; N = 10 control, 11 YM90K^DART^). **(G)** Schematic of the viral strategy used to label SPNs and astrocytes. Drd1a-Cre and Adora2a-Cre mice were transduced with AAV5-hSyn-DIO-GFP to label direct-pathway SPNs (dSPNs) and indirect-pathway SPNs (iSPNs), respectively, together with AstroHTP, which expresses HaloTag protein (HTP) and NLS-dTomato in astrocytes. GFP-positive SPNs located near NLS-dTomato-positive astrocytes were patch-clamped (held at −70 mV) to measure evoked excitatory postsynaptic currents (eEPSCs). **(H)** Representative image of a GFP-positive iSPN adjacent to an NLS-dTomato-positive, HTP-expressing astrocyte capturing Alexa647^DART^ (grey). Scale bar, 10 μm. **(I)** Slice pharmacology conditions and recording paradigms for panels J-O and P-U. *Pharmacology:* control, 300 nM Blank^DART^ + 300 nM YM90K^PEG36^ + 30 nM Alexa647^DART^; YM90K^DART^, 300 nM YM90K^DART^ + 30 nM Alexa647^DART^. *AMPAR-mediated eEPSCs (J–O):* recorded in the presence of gabazine and CPP to block GABA_A_ receptor- and NMDA receptor-mediated currents, respectively, isolating the AMPAR component of the eEPSC. *Glutamatergic eEPSCs (P-U):* recorded in gabazine alone, with CPP omitted, so NMDA receptor-mediated currents remain intact alongside the AMPAR component. **(J–O)** Astrocyte-tethered YM90K^DART^ does not act transcellularly on neuronal AMPARs. **(J)** Time course of normalized eEPSC amplitude in dSPNs. In the presence of CPP, YM90K^DART^-treated cells (green) showed no change relative to control (grey). Data are mean ± SEM. **(K)** Representative eEPSC traces from dSPNs at baseline (grey) and after treatment with control (black) or YM90K^DART^ (green). **(L)** End-point comparison of dSPN eEPSC amplitude (CPP condition): control 99.3 ± 12.6%; YM90K^DART^ 96.8 ± 15.5% (Mann–Whitney test, P = 0.5054). Control, 8 cells, 5 mice; YM90K^DART^, n = 8 cells, 5 mice. **(M)** Time course of normalized eEPSC amplitude in iSPNs. In the presence of CPP, YM90K^DART^ treated cells (green) showed no change relative to control (grey). Data are mean ± SEM. **(N)** Representative eEPSC traces from iSPNs at baseline (grey) and after treatment with control (black) or YM90K^DART^ (green). **(O)** End-point comparison of iSPN eEPSC amplitude: Control 93.5 ± 7.63%; YM90K^DART^ 94.6 ± 6.94% (Mann–Whitney test, P = 0.6947). Control, n = 12 cells, 6 mice; YM90K^DART^, n = 11 cells, 6 mice. (**P-U)** Astrocyte-specific inhibition of AMPARs reduces excitatory synaptic activity of SPNs. **(P)** Time course of normalized dSPN eEPSC amplitude; YM90K^DART^ (blue) showed a progressive reduction compared with control (grey). Data are mean ± SEM. **(Q)** Representative eEPSC traces from dSPNs at baseline (grey) and after treatment with control (black) or YM90K^DART^ (blue). **(R)** End-point comparison of dSPN eEPSC amplitude: control 108 ± 8.6%; YM90K^DART^ 62 ± 9.59% (Mann–Whitney test, P = 0.0029). Control, n = 10 cells, 8 mice, YM90K^DART^, n = 10 cells, 7 mice. **(S)** Time course of normalized eEPSC amplitude in iSPNs; YM90K^DART^ (blue) showed a progressive reduction compared with control (grey). Data are mean ± SEM.**(T)** Representative eEPSC traces from iSPNs at baseline (grey) and after treatment with control (black) or YM90K^DART^ (blue). **(U)** End-point comparison of iSPN eEPSC amplitude: control 93.9 ± 14.4%; YM90K^DART^ 48.0 ± 6.93% (Mann–Whitney test, P = 0.0103). Control n = 8 cells, 7 mice, YM90K^DART^ n = 15 cells, 8 mice.

**Figure 4.**
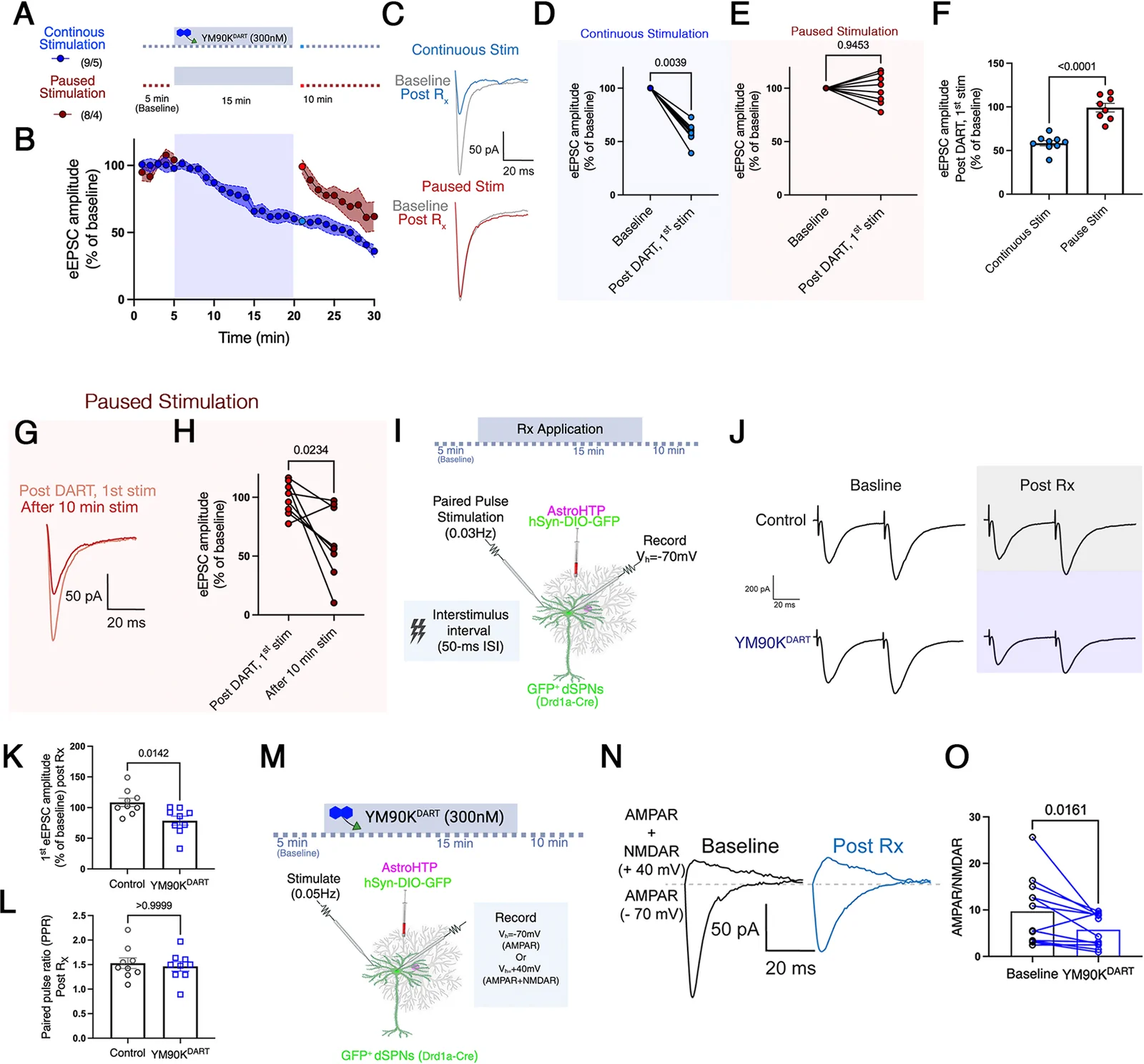
Astrocyte-specific AMPAR inhibition induces use-dependent postsynaptic LTD. **(A-H)** Astrocyte-specific inhibition of AMPARs reduces excitatory synaptic activity in dSPNs in an activity-dependent manner. **(A)** Recording paradigm for panels B-F. eEPSCs were recorded as described in Figure 3. Both groups received YM90K^DART^ (300 nM). In the continuous-stimulation group, synaptic stimulation continued throughout the baseline period, YM90K^DART^ application, and washout. In the paused-stimulation group, stimulation was suspended at the end of a 5-min baseline period, remained off during YM90K^DART^ application, and resumed after 15 min of drug exposure. **(B)** Time course of normalized eEPSC amplitude in dSPNs. The continuous-stimulation group (blue) exhibited a robust reduction in eEPSC amplitude during continuous synaptic stimulation. In contrast, the paused-stimulation group (red) showed stable eEPSC amplitudes immediately following resumption of stimulation after YM90K^DART^ exposure, followed by a progressive decrease with continued synaptic stimulation. Data are mean ± SEM. Continuous stimulation, n = 9 cells, 5 mice; paused stimulation, n = 8 cells, 4 mice**. (C)** Representative eEPSC traces from dSPNs at baseline (grey) and after YM90K^DART^ treatment with continuous stimulation (blue) or paused stimulation (red). **(D)** Paired comparison of eEPSC amplitude at baseline and after 15 min YM90K^DART^ application with continuous stimulation, 58.5 ± 3.01% (Wilcoxon test, P = 0.0039), n=9 cells, 5 mice. **(E)** Paired comparison of eEPSC amplitude at baseline and after 15 min YM90K^DART^ application with paused stimulation, 99.1 ± 4.93% (Wilcoxon test, P = 0.9453), n=8 cells, 4 mice. **(F)** Comparison of the first eEPSC amplitude after 15 min of drug application between the continuous- and paused-stimulation groups: continuous 58.5 ± 3.01%; paused 99.1 ± 4.93% (Mann–Whitney test, P < 0.0001). Continuous stimulation, n = 9 cells, 5 mice; paused stimulation, n = 8 cells, 4 mice**. (G)** Representative eEPSC traces from a dSPN during the paused-stimulation protocol, first after stimulation resumed (light red) and 10 minutes after continuous stimulation resumed (dark red). **(H)** Paired comparison of eEPSC amplitude before versus after the stimulation pause: first stimulation, 99.1 ± 4.93%; after 10 min of resumed stimulation, 62.0 ± 10.9% (Wilcoxon test, P = 0.0234), n = 8 cells, 4 mice**. (I-L)** Astrocyte-specific inhibition of AMPARs does not change presynaptic release probability. **(I)** Stimulation and recording paradigm for paired-pulse ratio (PPR) measurements, applied throughout panels J-K. Paired-pulse stimuli were delivered at 0.03 Hz, with a 20 Hz (50 ms) interstimulus interval. Pharmacology; control: 300 nM Blank^DART^ + 300 nM YM90K^PEG36^ + 30 nM Alexa647^DART^; YM90K^DART^: 300 nM YM90K^DART^ + 30 nM Alexa647^DART^. **(J)** Representative PPR traces in control and YM90K^DART^-treated conditions. Control traces are shown at baseline and post-treatment (black); YM90K^DART^-treated traces are shown at baseline and post-treatment (blue). **(K)** Quantification of the first eEPSC amplitude in the PPR experiment, normalized to baseline. YM90K^DART^ treatment reduced eEPSC amplitude (control 108 ± 6.77% of baseline YM90K^DART^ 78.6 ± 7.25% of baseline; Mann–Whitney test, P = 0.0142). **(L)** Quantification of PPR, showing no change following YM90K^DART^ treatment (control 1.53 ± 0.109; YM90K^DART^ 1.46 ± 0.0982; Mann–Whitney test, P > 0.9999). Both measures: n = 9 cells from 5 mice. **(M-O)** Astrocyte-specific inhibition of AMPARs reduces postsynaptic AMPAR signaling. **(M)** Recording paradigm for AMPAR/NMDAR ratio measurements characterized in N-O. **(N)** Representative AMPAR/NMDAR traces recorded at −70 mV (AMPAR) and +40 mV (NMDAR) in dSPNs, at baseline (black) and post- YM90K^DART^ (blue). **(O)** Quantification of AMPAR/NMDAR ratio at baseline and after YM90K^DART^ treatment: baseline 9.69 ± 2.05; post-Rx 5.74 ± 1.01 (Wilcoxon matched-pairs signed-rank test, P = 0.0161; n = 12 cells, 5 mice).

**Figure 5.**
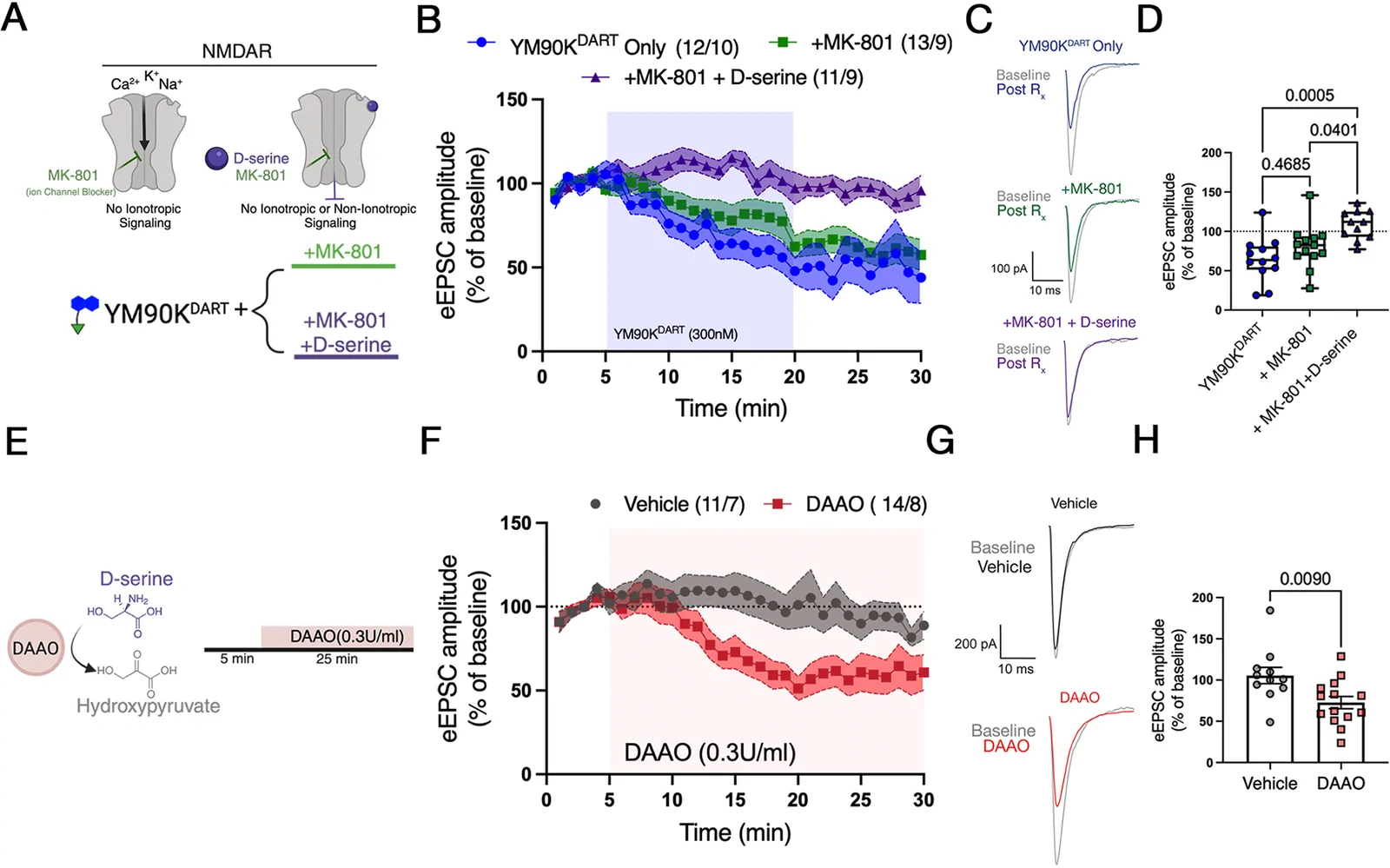
Astrocyte-specific inhibition of AMPARs reduces excitatory synaptic activity via non-ionotropic NMDAR signaling. **(A)** Schematic of the eEPSC recording paradigm used in panels B-D. Whole-cell dSPN recordings were performed at −70 mV in the presence of gabazine alone, gabazine plus MK-801 (to block ionotropic NMDAR activity), or gabazine plus MK-801 and D-serine (to inhibit both ionotropic and non-ionotropic NMDAR signaling). Astrocytic AMPARs were inhibited by bath application of YM90K^DART^ (300 nM). **(B)** Time course of normalized eEPSC peak amplitude in dSPNs. YM90K^DART^ alone (blue) induced a progressive reduction in eEPSC amplitude that persisted in the presence of MK-801 (green) but was abolished by co-application of MK-801 and D-serine (purple). Data are mean ± SEM (YM90K^DART^, 12 cells from 10 mice; YM90K^DART^ + MK-801, 13 cells from 9 mice; YM90K^DART^ + MK-801 + D-serine, 11 cells from 9 mice). **(C)** Representative eEPSC traces recorded at baseline (grey) and after YM90K^DART^ alone (blue), YM90K^DART^ + MK-801 (green), or YM90K^DART^ + MK-801 + D-serine (purple). **(D)** End-point eEPSC amplitude (10–20 min post-infusion) differed across conditions (Kruskal–Wallis test, P = 0.0007). Dunn's post hoc comparisons revealed no difference between YM90K^DART^ and YM90K^DART^ + MK-801 (P = 0.4685), but significant differences between YM90K^DART^ and YM90K^DART^ + MK-801 + D-serine (P = 0.0005), and between YM90K^DART^ + MK-801 and YM90K^DART^ + MK-801 + D-serine (P = 0.0401). **(E-H)** D-serine depletion reduces excitatory synaptic activity in dSPNs. **(E)** Schematic of the eEPSC recording paradigm used in panels F-H. Recordings were performed at −70 mV in the presence of gabazine. D-serine depletion was achieved by bath application of D-amino acid oxidase (DAAO, 0.3 U/ml). **(F)** Time course of eEPSC amplitude in dSPNs showing that DAAO (red) reduced eEPSC amplitude relative to vehicle (grey). Data are mean ± SEM (vehicle, 11 cells from 7 mice; DAAO, 14 cells from 8 mice). **(G)** Representative eEPSC traces at baseline (grey) and after vehicle (black) or DAAO (red). **(H)** End-point eEPSC amplitude (10–20 min post-infusion) was reduced in DAAO-treated slices compared with vehicle (vehicle 105 ± 9.96%; DAAO 72.7 ± 7.40%; Mann–Whitney test, P = 0.0090).

## Data Availability

All data and custom code are available at DOI: https://doi.org/10.70106/r4583. Further information and requests for resources, reagents, data, and code should be directed to and will be fulfilled by the corresponding author.
