# Supplementary material for: An astrocytic AMPAR-dependent parity signal preserves synaptic strength
Source: bioRxiv. 2026 Oct 11:2026.10.08.757679. Preprint. [Version 1] doi: 10.64898/2026.10.08.757679 (PMC13692175; doi:10.64898/2026.10.08.757679)
Supplement: Supplementary file 1 [file NIHPP2026.10.08.757679v1-supplement-2.pdf]

bioRxiv preprint doi: https://doi.org/10.64898/2026.10.08.757679; this version posted October 11, 2026. The copyright holder for this preprint (which was not certified by peer review) is the author/funder, who has granted bioRxiv a license to display the preprint in perpetuity. It is made available under a CC-BY 4.0 International license.

Supplementary Materials

Materials and Methodes

Animals

Mice aged 6–16 weeks were used for all experiments. Wild-type C57BL/6J mice, as well as transgenic Drd1a-cre [B6.FVB(Cg)-Tg(Drd1a-cre) EY262Gsat/Mmucd] and Adora2a-Cre [B6.FVB(Cg)-Tg(Adora2a-cre) KG139Gsat/Mmucd]; mouse lines were used to selectively target striatal direct- and indirect-pathway medium spiny neurons, respectively. Mice were housed under standard conditions, with a 12-h light/dark cycle and ad libitum access to food and water. For behavioral experiments, mice were housed on a reverse 12h/12h light-dark cycle, and experiments were conducted during the animals' active phase between zeitgeber times (ZT) 14 and 20 under dim red-light conditions. All experimental procedures were approved by the Institutional Animal Care and Use Committee (IACUC) and conducted in accordance with NIH guidelines.

AAV Production

AAVs were produced as previously described (*66*). In brief, HEK 293T cells were transfected with pAd-ΔF6, serotype plasmid AAV PHP.AX, and AAV transfer plasmid. After 72 h, the cells were lysed in 15 mM NaCl and 5 mM Tris-HCl (pH 8.5) and incubated with 50 U/ml benzonase for 30 min at 37 °C. The cell lysate was then centrifuged at 4,500 rpm for 30 min at 4 °C. The supernatant containing AAV was added to the top of an iodixanol gradient (15%, 25%, 40%, and 60% iodixanol solutions, from top to bottom) and centrifuged using a Beckman Ti-70 rotor at 60,000 rpm for 100 min. The viral solution was extracted from the virus layer (between the 40% and 60% iodixanol layers) using a 24-gauge needle and a 5-ml syringe, then concentrated using a 100-kDa filter. Viral titers were measured by quantitative PCR using a linearized plasmid containing the viral genome as the standard (Addgene). In addition, AAV5-GfaABC1D-GCaMP6f and AAV5-hSyn-DIO-GFP were purchased from Addgene Viral Service. Some batches of AAV-PHP.AX–GfaABC1D–HTP–NLS-dTomato–4×6T were custom-produced and purchased from VectorBuilder Inc.

Viral Surgery

Adult mice (6–16 weeks old) were anesthetized with 2% isoflurane and secured in a stereotaxic instrument (Model 1900, Kopf Instruments) on a heating pad set to 37 °C to maintain body temperature. Extended-release buprenorphine (0.5 mg/kg) was given subcutaneously and lubricating ocular ointment was applied. The scalp was shaved and then cleaned with alternating iodine and ethanol swabs. An incision was made along the midline of the skull, with 0.25% bupivacaine applied to the edges of the incision, then the surface of the skull was cleaned with 3% hydrogen peroxide. The skull was then leveled

bioRxiv preprint doi: https://doi.org/10.64898/2026.10.08.757679; this version posted October 11, 2026. The copyright holder for this preprint (which was not certified by peer review) is the author/funder, who has granted bioRxiv a license to display the preprint in perpetuity. It is made available under a CC-BY 4.0 International license.

along the anterior/posterior and medial/lateral axes. Craniotomies were drilled at desired coordinates with an Alpen carbide drill bit (R1001/4c, Coltene, Inc.) using a Midwest Tradition TL drill (780344, Dentsply Sirona). Phosphate buffered saline (PBS) with 200 mM NaCl (to prevent aggregation) was used to dilute AAVs. Virus was loaded into a glass micropipette (Wiretrol II 5 µL, 5-000-2005, Drummond Scientific Company) and injected through pre-drilled craniotomies using a manual injector (MO-10, Narishige) at ~100 nL/min. For dSTR targeting, virus was delivered across multiple injection sites to ensure uniform coverage (120 nL per depth). After each injection, the pipette was left in place for 3 to 5 min before retraction. Coordinates relative to bregma were AP +0.05, ML −1.7, DV −2.8 and −2.3; AP +0.15, ML −2.7, DV −3.2 and −2.7; AP +0.85, ML −1.3, DV −3.0 and −2.5; and AP +0.95, ML −2.3, DV −3.2 and −2.7. For motor cortex injections, 150 nL virus was delivered per site at AP +2.2, ML −0.9, DV −1.3; AP +2.2, ML −1.6, DV −1.6; AP +1.5, ML −0.8, DV −1.3; and AP +1.5, ML −1.6, DV −1.7. For hippocampus injections 500 nL virus was delivered at one site, AP −2.5, ML −1.7, DV −2.3. DV measurements are relative to skull surface at bregma. For surgeries not involving cannula implantation, the incision was closed with absorbable sutures (5-0 Webcryl, Pivetal), and mice were monitored during recovery and kept on a heating pad. Mice were used for experiments at least 3 weeks after viral surgery to allow for expression and recovery.

Cannula Implantation

For experiments involving intracranial DART delivery, guide cannulas were implanted immediately after viral injection. For striatal infusion, single guide cannulas (26 GA, guide cannula length 2 mm, dummy cannula 1 mm projection, Protech International or RWD Life Science with matched specifications) were implanted centered at the injection site (AP +0.5, ML −1.9, DV −2.5, [from skull surface at bregma, DV from the tip of the dummy cannula]). For intracerebroventricular access, bilateral cannulas (26 GA, center-to-center 2 mm, guide cannula length 2 mm, dummy cannula projection 0 mm, Protech International) were implanted targeting the lateral ventricles (AP −0.75, ML ±1.0, DV −2.25, [from skull surface at bregma, DV from the tip of the dummy cannula]). After slowly lowering cannulas, they were initially fixed in place using UV-curing Optibond (29669, Kerr Dental). A custom 3D-printed ‘headbar’ (*67*) was placed on the skull and secured in place with dental cement (Teets Cold Cure Denture Material powder and liquid, Coral, Co-Oral-Ite Dental Mfg.). For co-housed mice, an additional custom 3D-printed ‘headhat’ (*67*) was secured to the headbar to protect the cannula. Mice were used for experiments at least 3 weeks after viral surgery, allowing for expression and recovery.

Intracranial DART Delivery

DART infusions were performed in awake animals, head-fixed via the implanted headbar, using an automated syringe pump (PHD2000, Harvard Apparatus) connected to a 5 µL syringe (Model 75 RN, Hamilton) and a metal internal cannula (Protech International or RWD Life Science) extending 1.5 mm beyond the guide cannula for the striatum and 0.5

bioRxiv preprint doi: https://doi.org/10.64898/2026.10.08.757679; this version posted October 11, 2026. The copyright holder for this preprint (which was not certified by peer review) is the author/funder, who has granted bioRxiv a license to display the preprint in perpetuity. It is made available under a CC-BY 4.0 International license.

mm beyond the guide cannula for the lateral ventricle. Internal cannulas were connected to the syringe via PE50 tubing (0.023'' ID × 0.050'' OD, Protech International). Before infusion, the dummy cannula was removed, and the internal cannula was inserted. A total volume of 0.9 µL was infused at a rate of 0.1 µL/min, allowing an additional 6 min for diffusion before removing the internal cannula and replacing it with the dummy cannula. All DART reagents were prepared in Alzet ACSF. For infusions of YM90K^{DART} or YM90K^{PEG36}, a plastic internal cannula (PEEK, Protech International) was used to avoid potential reductions in the nitroaromatic structure of YM90K caused by metal. For all other infusions, a metal internal cannula was used.

To visualize DART ligand capture *in vivo*, we infused Alexa Fluor 647 ^{DART} $^($3µM, 0.9 µL total for 15 minutes) together with either control cocktail containing 30 µM untetherable YM90K^{PEG36} and 30 µM Blank^{DART} or 30µM YM90K^{DART} into the dSTR via an indwelling cannula and performed histology 24 hours later.

Histology and Immunostaining

Tissue processing for histology was performed using two protocols, reflecting the standard procedures of the laboratories where the analyses were conducted. The protocol for each experiment is indicated below.

Cryostat sections. Mice were anesthetized with tribromoethanol (200 mg/kg, Avertin) and transcardially perfused with TBS containing heparin (10 units/mL), followed by 4% paraformaldehyde (PFA) in TBS. Brains were dissected and post-fixed in 4% PFA overnight at 4 °C, cryoprotected in 30% sucrose, frozen in a 2:1 mixture of 30% sucrose and O.C.T. (TissueTek), and stored at −80 °C. Coronal sections (40–50 µm) were cut on a cryostat (CM3050S, Leica), collected as free-floating sections, and stored in a 1:1 mixture of TBS and glycerol at −20 °C until use.

Vibratome sections (Alexa647^{DART} capture). Mice were deeply anesthetized by isoflurane inhalation in a closed chamber with physical separation from the anesthetic until cessation of breathing and loss of response to toe and tail pinch, then transcardially perfused with PBS followed by 4% PFA in PB. Brains were dissected, post-fixed in 4% PFA overnight, then washed in PBS. Brains were embedded in 4% agarose, then coronal sections (50 µm) were cut on a vibratome (VT1200S, Leica). Alternating sections were mounted onto glass slides or collected as free-floating sections and stored in PBS at 4 °C until use. For experiments visualizing native fluorescence only (e.g., Alexa647^{DART} capture), slides were air-dried, rehydrated in PBS, rinsed with water, then coverslipped with Vectashield Vibrance Antifade Mounting Medium with DAPI (Vector Labs) and allowed to cure overnight at room temperature. Slides were imaged on an Olympus FV3000 confocal microscope or an Olympus VS200 Slide Scanner with filter cubes for DAPI, TRITC, and Cy5. For experiments requiring antibody labeling, sections were processed as described under 'Immunostaining' below.

bioRxiv preprint doi: https://doi.org/10.64898/2026.10.08.757679; this version posted October 11, 2026. The copyright holder for this preprint (which was not certified by peer review) is the author/funder, who has granted bioRxiv a license to display the preprint in perpetuity. It is made available under a CC-BY 4.0 International license.

Immunostaining. The following protocol was applied to both free-floating cryostat and vibratome sections. Sections were blocked in PBS containing 0.1% Triton X-100 and 10% normal goat serum (NGS) for 1–2 h at room temperature, then incubated with primary antibodies (Sox9 1:2000; Olig2 1:500; NeuN 1:1000; see Key Resources Table for list) diluted in the same buffer for 1–2 days at 4 °C. Sections were then incubated with Alexa Fluor 488-, 568-, or 647-conjugated secondary antibodies (1/250, see Key Resources Table for list) diluted in the same buffer for 2 h at room temperature. Sections were mounted in Vectashield Vibrance Antifade Mounting Medium with Dapi (Vector Labs). Confocal images were acquired on an Olympus FV3000 or Leica SP8 microscope with filter cubes for DAPI, FITC, TRITC, and Cy5.

HaloTag Surface Turnover

Pulse-chase experiment. Adult mice (8 weeks old) were stereotaxically injected with AAV.PHP.AX-GfaABC1D-HTP-NLSdTomato-4x6T (2×10^{12} vg/mL) into the dSTR and implanted with a guide cannula, as described above. Three weeks post-surgery, mice were head-fixed and received a unilateral intracranial infusion of 0.9 µL of 60 µM Biotin^{DART}. This pulse was followed, at intervals ranging from 1 to 72 h, by an infusion of 0.9 µL of 60 µM Alexa647^{DART}. 24 h after the second infusion, brains were collected and processed for cryostat sectioning as described under "Histology and Immunostaining." Sections were incubated in streptavidin–Alexa Fluor 750 (2 µg/mL in TBS) for 1 h at room temperature with gentle agitation, then washed three times in TBS.

Image analysis and modeling. Fluorescence images were analyzed using a custom MATLAB pipeline. Briefly, striatal regions of interest (ROIs) were defined within each brain section, and a multi-scale background subtraction was applied to each channel using iterative Gaussian filtering with increasing kernel sizes, producing background-subtracted intensity images. Astrocyte cell bodies were segmented using a difference-of-Gaussians approach applied to the ^{NLS}dTomato channel, followed by morphological opening to remove noise. Segmented objects were filtered by size and intensity to retain only high-quality astrocyte nuclei. For each segmented cell, mean pixel intensities were extracted from the Streptavidin-750 and Alexa647^{DART} channels across both signal and background pixel populations. Per-section background-subtracted intensity values were computed by subtracting the mean background pixel intensity from the mean signal pixel intensity for each channel. To normalize each channel to its maximum, background-subtracted intensities were divided by the maximum of an independently-fit single exponential (y-intercept for Streptavidin-750, fit to $y = A \cdot \exp(-k \cdot t)$, plateau for Alexa647^{DART}, fit to $y = A \cdot (1 - \exp(-k \cdot t))$), yielding values on a 0–1 scale. Normalized fluorescence ratios were then computed as 750/(750+647) for each time point. These ratios were fit to a biphasic exponential decay model, $y = A \cdot \exp(-k_1 \cdot t) + (1-A) \cdot \exp(-k_2 \cdot t)$, constrained such that $y(0) = 1$ (all surface HTP biotin-labeled), $y(\infty) = 0$ (all surface HTP replaced by newly synthesized protein), and $k_1 + k_2 = 1\ h^{-1}$. This constraint reduced the model to two free parameters: A (the fractional amplitude of the fast component) and k_1 (the fast rate constant), with $k_2 = 1$

bioRxiv preprint doi: https://doi.org/10.64898/2026.10.08.757679; this version posted October 11, 2026. The copyright holder for this preprint (which was not certified by peer review) is the author/funder, who has granted bioRxiv a license to display the preprint in perpetuity. It is made available under a CC-BY 4.0 International license.

- k_1. Model fitting was performed by nonlinear least squares (MATLAB *lsqcurvefit*). Component half-lives were calculated as $t½ = \ln(2)/k$, and an effective half-life was defined as the time at which the fitted curve crossed $y = 0.5$. Uncertainty was estimated by residual bootstrap resampling (10,000 iterations), with 95% confidence intervals derived from the 2.5th and 97.5th percentiles for each fitted parameter and the effective half-life.

Electron Microscopy

Adult mice (8 weeks old) were stereotaxically injected with AAV.PHP.AX-GfaABC1D-HTP-NLSdTomato-4x6T (2×10^{12} vg/mL) into the dSTR and implanted with a guide cannula, as described above. Three weeks post-surgery, mice were head-fixed and received a unilateral intracranial infusion of 0.9 µL of 30 µM Biotin^{DART}. 4 h after infusion, mice were deeply anesthetized by isoflurane inhalation in a closed chamber with physical separation from the anesthetic until cessation of breathing and loss of response to toe and tail pinch, then transcardially perfused for ultrastructural preservation. Perfusion was performed using a peristaltic pump at a slow flow rate (~6.75 mL/min), initially with 0.1 M sodium cacodylate buffer (pH 7.4), followed by ~30 mL freshly prepared 4% PFA in 0.075 M sodium cacodylate buffer (pH 7.4) with 2 mM $CaCl_2$. Brains were dissected and post-fixed overnight at 4 °C. The following day, brains were sectioned on a vibratome, and 50 µm coronal sections containing the region of interest were collected. Free-floating sections were incubated with streptavidin-Alexa Fluor 647 FluoroNanogold (Nanoprobes) for 2 h at room temperature to visualize Biotin^{DART} labeling, washed extensively, then briefly mounted onto glass slides and coverslipped with water for rapid fluorescence imaging on a slide scanner to localize Biotin^{DART}-positive regions. Coverslips were then carefully removed, and sections were transferred to a cryoprotectant solution (0.25 M sucrose, 7 mM $MgCl_2$, 50% glycerol in 0.05 M sodium cacodylate buffer) and stored at −20 °C until further processing. Prior to ultrastructural processing, sections were retrieved from cryoprotectant and washed to remove glycerol. Sections selected for ultrastructural analysis were then further fixed in 2.5% glutaraldehyde in PBS, washed three times for 10 min each in 0.15 M sodium cacodylate buffer, and subsequently washed three times for 10 min each in distilled water. Regions of interest were punched out from the vibratome sections, and silver enhancement was tested at 5, 30, 45, or 60 min; 45 min provided optimal labeling and was used for all subsequent analyses. Following silver enhancement, samples were washed six times in distilled water (5 min each) and stained overnight at 4 °C with 1% uranyl acetate in water. Tissues were then dehydrated through a graded ethanol series, infiltrated with resin, and polymerized. Ultrathin sections were cut perpendicular to the original vibratome sections to distinguish the positive signal from background and preserve anatomical orientation and local labeling patterns. Sections were imaged on a Thermo Fisher T12 transmission electron microscope operated at 80 kV using a Gatan OneView 4k × 4k camera. The majority of images were acquired at 13,000× magnification.

bioRxiv preprint doi: https://doi.org/10.64898/2026.10.08.757679; this version posted October 11, 2026. The copyright holder for this preprint (which was not certified by peer review) is the author/funder, who has granted bioRxiv a license to display the preprint in perpetuity. It is made available under a CC-BY 4.0 International license.

Ex-Vivo 2-Photon Calcium Imaging

Slice preparation. For 2P imaging, adult mice (8 weeks old) were stereotaxically injected with 1 µL of a mixture of AAV.PHP.AX-GfaABC1D-HTP-^{NLS}dTomato-4x6T (2 × 10^{12} vg/mL) and AAV5-GfaABC1D-GCaMP6f (7×10^{12} vg/mL) unilaterally into the dSTR (AP +0.5, ML −1.9, DV −2.8 and −2.3). 3−4 weeks post-injection, mice were deeply anesthetized with 200 mg/kg tribromoethanol (Avertin) and rapidly decapitated. The brain was quickly removed and placed in an aerated (95% O_2, 5% CO_2), ice-cold slicing solution containing: 125 mM NaCl, 2.5 mM KCl, 1.25 mM NaH_2PO_4, 25 mM $NaHCO_3$, 10 mM glucose, 0.4 mM L-ascorbic acid, 2 mM Na-pyruvate, 3 mM $MgCl_2$, and 0.1 mM $CaCl_2 \cdot 2H_2O$ (pH 7.3−7.4, 310 mOsm/kg). Using a razor, the cerebellum and olfactory bulbs were removed, and the cerebellum side of the brain was glued using cyanoacrylate glue (Krazy Glue, Elmer's) onto the vibratome (VT1200S, Leica) platform. Once the glue had sufficiently dried, the brain was quickly hemisected with a razor and submerged in an ice-cold slicing solution in the vibratome chamber. Coronal dSTR slices (350 µm thick) were cut with the vibratome in the same ice-cold solution. The hemisphere containing the viral infection was collected and immediately transferred into a standard artificial cerebrospinal fluid (ACSF) solution (33 °C, continuously aerated with 95% O_2, 5% CO_2) containing: 125 mM NaCl, 2.5 mM KCl, 1.25 mM NaH_2PO_4, 25 mM $NaHCO_3$, 10 mM glucose, 0.4 mM L-ascorbic acid, 2 mM Na-pyruvate, 1 mM $MgCl_2$, and 1 mM $CaCl_2 \cdot 2H_2O$. Slices were incubated at 33 °C for 30 min, then cooled to room temperature until imaging.

Image acquisition. Acute slices were transferred to a circulating perfusion bath driven by a peristaltic pump (120S, Watson-Marlow) delivering continuously aerated (95% O_2, 5% CO_2) ACSF maintained at 33 °C via an inline temperature controller (Perfusion Temperature Controller, Scientifica). Images were acquired on an upright two-photon microscope (Bruker) equipped with a tunable laser (InSight, Spectra Physics), with beam intensity modulated by a Pockels cell and scanning performed by linear galvanometers. Imaging was performed using an Olympus XLUMPLFLN 20× water-immersion objective (numerical aperture 1.00, working distance 2.0 mm; Olympus). Imaging was controlled by PrairieView software (Bruker). GCaMP6f was excited at 920 nm, and ^{NLS}dTomato-expressing astrocytes were identified by cross-excitation of dTomato with the same 920 nm laser; emission was collected through a 1040/920 filter. Images were acquired at 512 × 512 pixels with a pixel size of 0.271 µm, dwell time of 1.2 µs, 2× optical zoom, and a frame interval of 0.606 s/frame.

For each slice, experiments proceeded as follows. For experiments measuring spontaneous activity, a 10 min baseline was recorded in standard ACSF prior to tetrodotoxin (TTX) application. TTX (0.5 µM) was added to the bath, and slices were incubated for at least 10 min to block neuronal action potentials before further recordings. For experiments not requiring a spontaneous-activity baseline, TTX (0.5 µM) was present in the bath solution before slice transfer, and slices were incubated for 10 min before imaging. Fields of view were selected in the dorsolateral striatum on the basis of ^{NLS}dTomato and GCaMP6f co-expression. Following TTX equilibration, a 2 min baseline was acquired, after which AMPA (100 µM) and cyclothiazide (CTZ, 100 µM) were added to the bath; a 10 min video was acquired starting 90 s after the drug addition to account for tubing dead volume and

bioRxiv preprint doi: https://doi.org/10.64898/2026.10.08.757679; this version posted October 11, 2026. The copyright holder for this preprint (which was not certified by peer review) is the author/funder, who has granted bioRxiv a license to display the preprint in perpetuity. It is made available under a CC-BY 4.0 International license.

bath equilibration. Between slices, the bath was thoroughly washed with ACSF for 10−15 min.

ROI-based analysis. Evoked AMPA/CTZ responses were analyzed using a manual ROI-based approach in Fiji and MATLAB. Astrocytes were manually outlined in Fiji using the polygon tool and added to the ROI manager. Mean pixel values per frame were extracted for each ROI and imported into MATLAB for further analysis. ΔF/F was calculated as $(F-F_0)/F_0$, where F_0 was defined as the mean fluorescence in the first 30 s of the 2 min baseline acquisition, prior to drug arrival in the imaging chamber. Traces were cropped to 5 min to capture the majority of the drug response. Peak response was defined as the maximum ΔF/F value within the cropped trace. The area under the curve (AUC) was calculated by numerical integration using trapezoidal method (MATLAB function).

AQuA2 event detection. Spontaneous astrocytic calcium activity was analyzed using AQuA2 (*21*), an event-based detection framework that identifies spatiotemporal calcium events directly from fluorescence dynamics without predefined ROIs. Raw TIFF stacks were imported into AQuA2 (run in MATLAB R2022b) and processed using the default parameter set, which was applied uniformly across all datasets and conditions to avoid parameter-dependent bias. AQuA2's built-in pipeline automatically performed preprocessing, spatiotemporal event segmentation, and feature quantification. Per-event features, including event duration, amplitude, frequency, rise time, and other dynamic characteristics were exported for downstream analysis in custom Python scripts.

Cell Type Specificity Quantification

Coronal sections from dSTR of AstroHTP-injected mice were immunostained for Sox9, NeuN, and Olig2 and imaged on an Olympus FV3000 confocal microscope using identical acquisition settings across samples. Confocal images were analyzed using ilastik-generated pixel classification maps. Briefly, an ilastik (v1.4.0) Pixel Classification project was trained on n = 5 representative training images using the default feature set. RFP^+ nuclei were hand-annotated as the foreground class, while the background was annotated across multiple distinct images to capture variability in tissue autofluorescence and non-specific signal. The trained classifier was then applied uniformly to all images in the dataset to generate probability maps. Pixel classification maps were processed using a custom Fiji macro (Synbot (*68*)) that batch-processed all images within a user-selected directory. For each image, the probability map was thresholded into a binary mask for each class, and watershed segmentation was applied to separate adjacent or touching objects. Segmented objects were counted using the Analyze Particles function with a minimum size threshold of 70 $pixels^2$ and exclusion of border-touching objects. Per-class counts and image filenames were compiled into a results table and exported as a CSV file for downstream analysis, including colocalization analyses between $^{NLS}dTomato^+$ and $Sox9^+/NeuN^+/Olig2^+$ nuclei to assess cell-type specificity.

bioRxiv preprint doi: https://doi.org/10.64898/2026.10.08.757679; this version posted October 11, 2026. The copyright holder for this preprint (which was not certified by peer review) is the author/funder, who has granted bioRxiv a license to display the preprint in perpetuity. It is made available under a CC-BY 4.0 International license.

Open-Field Behavioral and Kinematic Analysis

Behavioral paradigm. Adult mice (8–16 weeks old) were stereotaxically injected with AAV.PHP.AX-GfaABC1D-HTP-NLSdTomato-4x6T (2×10^{12} vg/mL) into the dSTR and implanted with a guide cannula, as described above. Three weeks post-surgery, mice were head-fixed and received a unilateral intracranial infusion of 0.9 µL of either: (1) 30 µM YM90K^{DART} + 3 µM Alexa647^{DART}, or (2) 30 µM YM90K^{PEG36} + 30 µM Blank^{DART} + 3 µM Alexa647^{DART} (control cocktail). 1 h and 24 h after DART infusions, mice were placed in the open field for 30 min. Following the second open field session, brains were collected and processed for vibratome sections as described under "Histology and Immunostaining."

Behavioral recording and pose estimation. Mice were recorded in a 12-inch-square elevated open-field arena with a transparent floor and walls, surrounded by angled mirrors. A single camera positioned beneath the arena captured five simultaneous views of the mouse (one direct view through the floor and four mirror reflections) at 90 frames per second (fps) during 30 min sessions. Three-dimensional pose estimation was performed using DANNCE (*22*) which tracked 18 body parts across all camera views. For subsequent behavioral quantification, 3D coordinates were projected onto the 2D bottom-view plane and converted from pixels to centimeters using a calibrated pixel resolution derived from known arena dimensions. A subset of tracked keypoints was used for subsequent analysis: the snout, spine midpoint (used as the body center), and tail base.

Tracking artifact removal. Occasional escape attempts caused mice to leave the field of view of the mirror reflections, producing transient tracking failures in which keypoints moved erratically to physically implausible positions at impossible speeds. To identify and remove these artifacts, frame-to-frame speed of the body center was computed, and frames in which speed exceeded 90 cm/s were flagged. This threshold was determined empirically from inspection of tracking data across recordings; normal mouse locomotion rarely approaches this speed. Flagged frames were expanded by a 10-frame buffer on each side (~0.11 s at 90 fps) to account for gradual tracking degradation surrounding each event. All six coordinate channels (body center, snout, and tail base, in x and y) were set to NaN at flagged frames. This ensured that all downstream metrics, including speed, turning, trajectory-based measures, and immobility, automatically excluded artifact-contaminated data.

Locomotion. Instantaneous speed was calculated as the frame-to-frame Euclidean displacement of the body center (spine midpoint) multiplied by the frame rate (90 fps), yielding units of cm/s. Speed values exceeding 90 cm/s were set to NaN as an additional safeguard against residual tracking noise propagated from frames adjacent to those excluded during artifact removal. Total distance traveled was computed within each 5 min bin by summing instantaneous displacements, excluding NaN frames; six bins were generated per 30 min session.

Turning analysis. The instantaneous heading direction of the mouse was defined as the angle from the tail base to the snout in the 2D plane. This heading angle was unwrapped to remove discontinuities at the ±180° boundary and low-pass filtered to reduce high-frequency noise. A complete turn was defined as a cumulative heading change of ≥360° in

bioRxiv preprint doi: https://doi.org/10.64898/2026.10.08.757679; this version posted October 11, 2026. The copyright holder for this preprint (which was not certified by peer review) is the author/funder, who has granted bioRxiv a license to display the preprint in perpetuity. It is made available under a CC-BY 4.0 International license.

a single direction without returning through the starting angle. To guard against spurious detections, candidate turns were rejected if the heading reversed by more than 180° from the cumulative turn trajectory before completing the 360° rotation, indicating the mouse changed direction mid-turn, or if any frame between the start and completion of the candidate turn contained a NaN (i.e., a tracking artifact). Turns were classified as leftward (counterclockwise from the perspective of the mouse) or rightward (clockwise from the perspective of the mouse) based on the sign of the cumulative heading change. Net turns per session were computed as the difference between rightward and leftward turn counts; total leftward and rightward turn counts were tallied separately.

Thigmotaxis. To quantify thigmotaxis, the proportion of time spent in the center versus the edge of the arena was computed from the body center trajectory. The center zone was a 12.5 × 12.5 cm square region centered on the midpoint between the 1st and 99th percentiles of the body center's x and y coordinates. This data-driven centering approach accounted for minor variations in tracking coordinate offsets across sessions; deviations greater than 10% of arena width from the geometric arena center were overridden by the geometric center. Center occupancy was expressed as the percentage of total frames in which the body center fell within the center zone; edge occupancy was the complement. Center occupancy was computed per 30 min session.

Immobility. Immobility was assessed using a bout-based analysis of smoothed locomotor speed. The instantaneous speed signal was smoothed with a 90-frame (1 s) moving median filter and log10-transformed. Frames in which the transformed speed fell below a threshold of 0 (corresponding to a raw speed of <1 cm/s) were classified as immobile. Contiguous immobile frames were grouped into bouts, and bouts shorter than 1 s (90 frames) were discarded to exclude brief pauses during active locomotion. From the remaining bouts, the total percent time immobile was computed per 30 min session.

Electrophysiology: Astrocyte-Targeted DART

Slice preparation. Young adult mice (6−8 weeks old) of either Drd1a-Cre or Adora2a-Cre lines were stereotaxically injected with 1 µL of a mixture of AAV.PHP.AX-GfaABC1D-HTP-^{NLS}dTomato-4x6T (2 × 10^{12} vg/mL) and AAV5-hSyn-DIO-GFP (2.3 × 10^{12} vg/mL) unilaterally into the dSTR (AP +0.5, ML −1.9, DV −2.8 and −2.3). 3-4 weeks post-injection, mice were deeply anesthetized with 200 mg/kg tribromoethanol (Avertin) and rapidly decapitated. Brains were quickly removed and placed in ice-cold, carbogenated (95% O2, 5% CO2) sucrose-based cutting solution containing (in mM): 3.3 KCl, 1.3 NaH_2PO_4, 26 $NaHCO_3$, 11 D-glucose, 211 sucrose, 0.5 $CaCl_2$, and 10 $MgCl_2$. Coronal slices (300 µm) containing the dSTR were cut on a vibratome (VT1000S, Leica). Slices were transferred to carbogenated ACSF and allowed to recover at 33 °C for 50 min, then for an additional 10 min at room temperature before recording. ACSF contained (in mM): 124 NaCl, 3.3 KCl, 1.3 NaH_2PO_4, 26 $NaHCO_3$, 11 D-glucose, 2 $CaCl_2$, and 1 $MgCl_2$, and was continuously equilibrated with 95% O_2 / 5% CO_2 during recovery and recording. Slices were used for up to 4 h after the end of the recovery period.

bioRxiv preprint doi: https://doi.org/10.64898/2026.10.08.757679; this version posted October 11, 2026. The copyright holder for this preprint (which was not certified by peer review) is the author/funder, who has granted bioRxiv a license to display the preprint in perpetuity. It is made available under a CC-BY 4.0 International license.

DART Application. To inhibit astrocytic AMPARs in acute slices, YM90K^{DART} was bath-applied during recording. Slices were continuously perfused with ACSF containing either 300 nM YM90K^{DART} or a control cocktail of 300 nM YM90K^{PEG36} (an untetherable variant lacking the HaloTag ligand) and 300 nM Blank^{DART} (a pharmacologically inert tethered ligand). In both conditions, 30 nM Alexa647^{DART} was included as a fluorescent tracer to verify ligand capture. For experiments measuring miniature EPSCs (mEPSC), slices were preincubated in the corresponding DART solution for at least 30 min before recording. For experiments measuring evoked EPSCs (eEPSC), a stable 5 min baseline was first acquired in standard ACSF, after which the DART solution was bath-applied for 15 min, followed by a 10 min washout period during which recordings continued.

Whole-cell recordings. Whole-cell voltage-clamp recordings were obtained from GFP$^+$ SPNs adjacent to ^{NLS}dTomato$^+$ astrocytes in a submerged recording chamber superfused with warmed ACSF (29–32 °C). Patch pipettes (2–4 MΩ) were pulled from borosilicate glass. For mEPSC recordings, pipettes were filled with an internal solution containing (in mM): 145 CsCl, 5 NaCl, 10 HEPES, 10 EGTA, 4 Mg-ATP, and 0.3 Na-GTP. For eEPSC recordings, the internal solution contained (in mM): 130 $CsMeSO_4$, 5 TEA-Cl, 8 NaCl, 10 HEPES, 0.5 EGTA, 4 Mg-ATP, 0.4 Na-GTP, 10 phosphocreatine disodium salt hydrate, and 3 QX-314. All internal solutions were adjusted to pH of 7.3–7.4 with CsOH and to an osmolality of 280–285 mOsm/kg. After formation of a giga-ohm seal and break-in, cells were voltage-clamped at −70 mV. Electrical stimulation (0.1 ms pulses) for evoked recordings was delivered every 20 s through a concentric bipolar electrode (FHC). For each cell, stimulus intensity was adjusted to evoke approximately 40% of the maximal eEPSC amplitude by gradual increase from threshold. Paired-pulse ratios (PPRs) were calculated as the amplitude of the second EPSC divided by that of the first EPSC, using a 50 ms interstimulus interval. Access resistance and holding current were monitored throughout each recording, and cells exhibiting instability were excluded from analysis.

For single pulse eEPSC recordings, stimuli were delivered every 20s. For paired-pulse recordings, stimulus pairs were delivered every 30s, with a 50-ms interstimulus interval (ISI) within each pair.

AMPAR component was measured as the peak current at −70 mV, whereas the NMDAR component was measured at +40 mV at the post-stimulus latency at which the AMPAR-mediated current recorded at −70 mV had decayed to baseline.

Pharmacology. For mEPSC recordings, ACSF contained tetrodotoxin (TTX, 1μM) to block action potentials and gabazine (SR-95531, 10 μM) to block $GABA_A$ receptor-mediated currents. For eEPSC recordings, ACSF contained gabazine to isolate excitatory transmission. To isolate AMPAR-mediated currents in experiments testing astrocyte selectivity, the NMDAR antagonist CPP (10 μM) was included in the bath. In experiments testing the requirement for ionotropic NMDAR conductance, the open-channel NMDAR blocker MK-801 (10 μM) was applied to the bath. To assess the contribution of NMDAR co-agonist availability, D-serine (10 μM) was co-applied with MK-801. To enzymatically deplete extracellular D-serine, D-amino acid oxidase (DAAO, 0.3 U/mL) was bath-applied together with catalase (300 U/mL) using the same recording paradigm as for eEPSCs. All

bioRxiv preprint doi: https://doi.org/10.64898/2026.10.08.757679; this version posted October 11, 2026. The copyright holder for this preprint (which was not certified by peer review) is the author/funder, who has granted bioRxiv a license to display the preprint in perpetuity. It is made available under a CC-BY 4.0 International license.

drugs were applied via the perfusion line and allowed to equilibrate in the recording chamber prior to or during recording, as described for each experiment in the results.

Data acquisition and analysis. Signals were acquired using a HEKA EPC 10 amplifier controlled by PATCHMASTER NEXT software (HEKA Elektronik). mEPSCs were recorded for 5 min per cell following a brief stabilization period after break-in. eEPSCs were recorded for up to 30 min per cell, and slices used for eEPSC recordings were not reused. For mEPSC quantification, the most stable 100 s segment from each recording was selected for analysis. Event detection and measurements were performed in FITMASTER NEXT (HEKA Elektronik) using consistent detection thresholds and criteria across cells. For eEPSC quantification, peak-to-anti-peak amplitudes were measured in PATCHMASTER NEXT. Representative traces were exported from FITMASTER NEXT and prepared for figures in Inkscape.

Electrophysiology: Neuron-Targeted DART

Slice preparation. Adora2a-Cre mice (8–10 weeks old) were stereotaxically injected with AAV10-CAG-DIO-HTP-2A-dTomato-WPRE (active HTP, 1.5×10^{12} vg/mL) or AAV10-CAG-DIO-ddHTP-2A-dTomato-WPRE (dead HTP control, 1.5×10^{12} vg/mL) at 80 nL per depth across multiple sites in the dSTR (AP +0.05 mm, ML ±1.7 mm, DV −2.8, −2.3, and −1.8 mm; AP +0.15 mm, ML ±2.5 mm, DV −3.1, −2.76, and −2.1 mm; AP +0.85 mm, ML ±1.3 mm, DV −3.0, −2.5, and −2.0 mm; and AP +0.95 mm, ML ±2.1 mm, DV −3.1, −2.6, and −2.1 mm, all relative to bregma). After 3–5 weeks for expression, mice were deeply anesthetized with isoflurane and decapitated. Coronal brain slices (300 µm) containing dSTR were cut on a Vibratome (Leica, VT1200S), in ice-cold high sucrose cutting solution containing (in mM): 220 sucrose, 3 KCl, 1.25 NaH2PO4, 25 $NaHCO_3$, 12 $MgSO_4.7H_2O$, 10 glucose, and 0.2 $CaCl_2$, bubbled with 95% O_2 / 5% CO_2. Slices were transferred to ACSF and allowed to recover at 33 °C for 40–60 min, then cooled to room temperature (22–24 °C) before recording. ACSF contained (in mM): 120 NaCl, 3.3 KCl, 1.23 NaH_2PO_4, 1 $MgSO_4$, 2 $CaCl_2$, 25 $NaHCO_3$, and 10 glucose (pH 7.3), continuously equilibrated with 95% O_2 / 5% CO_2.

DART Application. To test whether YM90K^{DART} inhibits neuronal AMPARs when tethered to neuronal HTP, the ligand was bath-applied to slices expressing either active HTP or catalytically inactive HTP (ddHTP, negative control). Slices were continuously perfused with ACSF containing 300 nM YM90K^{DART}; 30 nM Alexa647^{DART} was included as a fluorescent tracer to verify ligand capture. A stable 7.5 min baseline was first acquired in standard ACSF, after which the DART solution was bath-applied for 15 min, followed by a 7.5 min washout period during which recordings continued.

Whole-cell recordings. Whole-cell voltage-clamp recordings were obtained from dTomato^{+} SPNs in slices perfused with bubbled ACSF at 29–30 °C (2 mL/min). Patch pipettes (4–6 MΩ) were filled with an internal solution containing (in mM) 130 CsMeS, 1 $MgCl_2$, 0.5 EGTA, 10 HEPES, 4 Mg-ATP, 0.5 Na-GTP, 10 Na_2-phosphocreatine, and 4 QX-314, adjusted to pH 7.3 with CsOH and 290 mOsm. Cells were voltage clamped at –

bioRxiv preprint doi: https://doi.org/10.64898/2026.10.08.757679; this version posted October 11, 2026. The copyright holder for this preprint (which was not certified by peer review) is the author/funder, who has granted bioRxiv a license to display the preprint in perpetuity. It is made available under a CC-BY 4.0 International license.

70 mV. Electrical stimulation (0.3 ms pulses, 60–70% maximum response, 150–300 µA) was delivered every 30s through a stimulating electrode placed 60–100 µm from the recorded neuron. Series resistance was monitored using 5–10 mV hyperpolarizing steps interleaved with stimuli, and cells with series resistance changes greater than ~15% during the experiment were excluded.

Pharmacology. To isolate AMPAR-mediated EPSCs, picrotoxin (50 µM, $GABA_A$ receptor antagonist) and APV (10 µM, NMDAR antagonist) were included in the bath.

Data acquisition and analysis. Signals were acquired using a Multiclamp 700B amplifier (Molecular Devices), filtered at 10 kHz, and digitized using an Axon Digidata 1550B with pClamp 10.7 (Molecular Devices). Data were analyzed in Clampfit 10.7.

Quantification and statistical analysis

Reported values are mean ± SEM. Statistical analyses were performed in GraphPad Prism 10. Comparisons used Student's t-test (paired or unpaired), the Mann–Whitney test, the Wilcoxon matched-pairs signed-rank test, two-way ANOVA (with Fisher's LSD or Šídák's multiple-comparisons post hoc tests, as indicated), and the Kruskal–Wallis test (with Dunn's post hoc test), as specified in each figure legend. The statistical test, sample size, and P value are reported in each figure or figure legend. No statistical methods were used to predetermine sample size.

For *ex vivo* two-photon calcium imaging, multiple regions of interest (ROIs) or calcium events were recorded per slice and per animal; to avoid pseudoreplication, these measurements were aggregated at the slice level, and statistical comparisons were performed at the slice level as the unit of analysis. ROI, event, slice, and animal numbers are reported in the corresponding figure legends.

For two-photon experiments, conditions being compared (for example, $\text{Blank}^{\text{DART}}$ versus $\text{YM90K}^{\text{DART}}$) were interleaved and acquired side by side, using cage mates where possible, with groups balanced to minimize confounding differences. In vivo experiments used male mice only; where possible, cage mates were used, and groups were balanced to minimize confounding factors.

For whole-cell electrophysiology, the recorded cell was treated as the statistical unit, following standard practice for slice electrophysiology; cells were sampled across multiple mice for each condition, and the numbers of cells and mice are reported in each figure legend.

bioRxiv preprint doi: https://doi.org/10.64898/2026.10.08.757679; this version posted October 11, 2026. The copyright holder for this preprint (which was not certified by peer review) is the author/funder, who has granted bioRxiv a license to display the preprint in perpetuity. It is made available under a CC-BY 4.0 International license.

Antibodies

Reagent or Resource	Source	Identifier
Rabbit polyclonal anti-Sox9	Millipore	Cat# AB5535; RRID:AB_2239761
Mouse Monoclonal anti-NeuN Antibody, clone A60	Millipore	Cat# MAB377, RRID:AB_2298772
Mouse IgG2a monoclonal anti-Olig2, clone 211F1.1	Millipore	Cat# MABN50, RRID:AB_10807410
Streptavidin–FluoroNanogold 647	FluoroNanogold / Nanoprobes	Cat# 7516
Goat anti-mouse IgG2a Alexa Fluor 568	Thermo Fisher Scientific	RRID:AB_2535773
Goat anti-mouse IgG2b Alexa Fluor 568	Thermo Fisher Scientific	RRID:AB_2535780
Goat anti-mouse Alexa Fluor 488	Thermo Fisher Scientific	RRID:AB_2534057
Goat anti-rabbit Alexa Fluor 568	Thermo Fisher Scientific	RRID:AB_143157
Goat anti-rabbit Alexa Fluor 647	Thermo Fisher Scientific	RRID:AB_2921064
Donkey anti-mouse Alexa Fluor 647	Jackson ImmunoResearch	RRID:AB_162542
Donkey anti-rabbit Alexa Fluor 488	Jackson ImmunoResearch	RRID:AB_3107111

Chemicals, Peptides, and Drugs

Reagent or Resource	Source	Identifier
YM90K^{PEG36}	Tadross Lab	Custom synthesis
YM90K^{DART}	Tadross Lab	Custom synthesis

bioRxiv preprint doi: https://doi.org/10.64898/2026.10.08.757679; this version posted October 11, 2026. The copyright holder for this preprint (which was not certified by peer review) is the author/funder, who has granted bioRxiv a license to display the preprint in perpetuity. It is made available under a CC-BY 4.0 International license.

Reagent or Resource	Source	Identifier
Blank^{DART}	Tadross Lab	N/A
Alexa647^{DART}	Tadross Lab	N/A
Biotin^{DART}	Tadross Lab	N/A
D-Serine	Sigma-Aldrich	Cat# S4250-5
RS-AMPA	Tocris Bioscience	Cat# 0169
Cyclothiazide	Tocris Bioscience	Cat# 0713
NBQX	Tocris Bioscience	Cat# 0373
D-APV (AP5)	Tocris Bioscience	Cat# 0106/1
(R)-CPP	Tocris Bioscience	Cat# 0247
(+)-MK 801 maleate	Tocris Bioscience	Cat# 0924
Picrotoxin	Tocris Bioscience	Cat# 1128/1G
Tetrodotoxin (TTX)	Tocris Bioscience	Cat# 1078/1
Gabazine (SR-95531)	Tocris Bioscience	Cat# 1262
D-Amino Acid Oxidase (DAAO; porcine kidney)	Sigma-Aldrich	Cat# A5222-100UN
Catalase from bovine liver	Sigma-Aldrich	Cat# C1345-1G

Experimental Models: Organisms/Strains

Reagent or Resource	Source	Identifier
C57BL/6J mice	Jackson Laboratory	RRID:IMSR_JAX:000664

bioRxiv preprint doi: https://doi.org/10.64898/2026.10.08.757679; this version posted October 11, 2026. The copyright holder for this preprint (which was not certified by peer review) is the author/funder, who has granted bioRxiv a license to display the preprint in perpetuity. It is made available under a CC-BY 4.0 International license.

Reagent or Resource	Source	Identifier
Drd1a-Cre mice	GENSAT / Jackson Laboratory	RRID:IMSR_JAX:028298
Adora2a-Cre mice	MMRRC / Jackson Laboratory	RRID:IMSR_JAX:040186

Cell Lines

Reagent or Resource	Source	Identifier
One Shot™ Stbl3™ Chemically Competent E. coli	Thermo Fisher Scientific	Cat# C737303
HEK293T cells	ATCC	RRID:CVCL_0063

Recombinant DNA / Viral Vectors

Reagent or Resource	Source	Identifier
pZac2.1 GfaABC1D-cyto-GCaMP6f (AAV5)	Addgene	RRID:Addgene_52925
AAV-GfaABC1D-HTP-NLSdTomato-4×6T (PHP.AX)	Tadross Lab	Custom
AAV-hSyn-DIO-GFP (AAV5)	Addgene	RRID:Addgene_50457
AAV-CAG-DIO-HTP-2A-dTomato-WPRE (AAV10)	Tadross Lab	Custom
AAV-CAG-DIO-ddHTP-2A-dTomato-WPRE (AAV10)	Tadross Lab	Custom
CAG-CreON-HT2.0-IRES-dTomato-Farnesylated (AAV10)	Tadross Lab	Custom
pAAV-GfaABC1D-Cre-4x6T (PHP.eB)	Addgene	RRID:Addgene_196410
pAD-ΔF6	Addgene	RRID:Addgene_112867
pUC-mini-iCAP-PHP.eB	Addgene	RRID:Addgene_103005
pUCmini-iCAP-PHP.AX	Addgene	RRID:Addgene_195218

bioRxiv preprint doi: https://doi.org/10.64898/2026.10.08.757679; this version posted October 11, 2026. The copyright holder for this preprint (which was not certified by peer review) is the author/funder, who has granted bioRxiv a license to display the preprint in perpetuity. It is made available under a CC-BY 4.0 International license.

1163 **Software and Algorithms**

Reagent or Resource	Source	Identifier
Fiji (ImageJ)	NIH	RRID:SCR_002285
ilastik	Berg et al.	RRID:SCR_015246
Imaris	Bitplane / Oxford Instruments	RRID:SCR_007370
FITMASTER NEXT	HEKA Elektronik	N/A
PATCHMASTER NEXT	HEKA Elektronik	N/A
Clampfit v10.7	Molecular Devices	RRID:SCR_011323
PrairieView	Bruker	RRID:SCR_017142
MATLAB R2022b	MathWorks	RRID:SCR_001622
AQuA2	Wang et al. / GitHub	https://github.com/yu-lab-vt/AQuA2
DANNCE	Dunn et al.	https://github.com/spoonsso/dannce
Inkscape	Inkscape Project	RRID:SCR_014479
GraphPad Prism 10	GraphPad Software	RRID:SCR_002798
SynBot (Fiji plugin)	GitHub	https://github.com/cleterrier/SynBot

1164 **Other**

Reagent or Resource	Source	Identifier
PEEK guide cannula (C315G/PK, 2.5 mm)	Plastics One	Cat# C315G/PK
Internal cannula (C315I)	Plastics One	Cat# C315I
5 µl Hamilton syringe (75 RN)	Hamilton	Cat# 75 RN
PHD2000 syringe pump	Harvard Apparatus	Cat# PHD2000
Olympus FV3000 confocal microscope	Olympus	N/A

bioRxiv preprint doi: https://doi.org/10.64898/2026.10.08.757679; this version posted October 11, 2026. The copyright holder for this preprint (which was not certified by peer review) is the author/funder, who has granted bioRxiv a license to display the preprint in perpetuity. It is made available under a CC-BY 4.0 International license.

Reagent or Resource	Source	Identifier
Leica SP8 confocal microscope	Leica Microsystems	N/A
Olympus VS200 Slide Scanner	Olympus	N/A
Leica VT1200S vibratome	Leica Microsystems	Cat# VT1200S
VT-1000S vibratome	Leica Microsystems	Cat# VT-1000S
Bruker 2-photon microscope	Bruker	N/A
InSight tunable laser	Spectra Physics	N/A
Multiclamp 700B amplifier	Molecular Devices	Cat# 700B
HEKA EPC 10 amplifier	HEKA Elektronik	Cat# EPC 10
Digidata 1550B	Molecular Devices	Cat# 1550B
Thermo Fisher T12 transmission electron microscope	Thermo Fisher Scientific	Cat# T12
Gatan OneView 4k × 4k camera	Gatan	N/A
Tissue-Tek® O.C.T. Compound	Sakura® Finetek	Cat# 4583
Triton™ X-100 Surfact-Amps™ Detergent Solution	Thermo Fisher Scientific	Cat# 28314
VWR® Superfrost® Plus Micro Slide	VWR	Cat# 48311-703
Tween® 20 (Polysorbate), Molecular Biology Grade	Promega	Cat# H5151
Formaldehyde (37% by Weight/Molecular Biology)	Thermo Fisher Scientific	Cat# BP531-25

bioRxiv preprint doi: https://doi.org/10.64898/2026.10.08.757679; this version posted October 11, 2026. The copyright holder for this preprint (which was not certified by peer review) is the author/funder, who has granted bioRxiv a license to display the preprint in perpetuity. It is made available under a CC-BY 4.0 International license.

Figure S1. Supplement to Figure 1.

Further characterization of astrocyte-specific DART.

(A) AstroHTP-transduced striatal astrocytes capture DART ligands at perisynaptic processes. AstroHTP was injected into the dSTR of adult C57BL/6J mice. Three weeks post-injection, 30 µM biotin-conjugated HTP ligand ($\text{Biotin}^{\text{DART}}$) was infused into the striatum of head-fixed mice for 15 min. Coronal striatal sections were processed for immuno-electron microscopy and labeled with Alexa Fluor 647-conjugated FluoroNanogold™ to visualize HTP-bound DART ligands. Representative image from a coronal section of a C57BL/6J mouse brain expressing AstroHTP (magenta), 4 h post-infusion with $\text{Biotin}^{\text{DART}}$ and post-hoc labeling with Alexa Fluor 647-conjugated FluoroNanogold™ (grey). Magenta box, negative-control hemisphere (no virus injected); blue box, experimental hemisphere. Scale bars: 1000 µm, 10 µm.**(B)** Representative electron micrograph from a coronal striatal section 4 h after intracranial infusion of $\text{Biotin}^{\text{DART}}$, showing Alexa647-nanogold labeling of astrocytic processes (green). Blue outline, experimental hemisphere; magenta outline, control hemisphere. Scale bar, 100 nm. **(C–E)** AstroHTP can be used to target astrocytes in different brain regions. **(C)** AstroHTP was injected into the motor cortex or the hippocampus of adult C57BL/6J mice. Three weeks post-injection, 3 µM Alexa647-DART was infused into the ventricles of head-fixed mice for 15 min. **(D)** Representative images from a coronal section of an adult C57BL/6J mouse hippocampus and motor cortex, 24 h post-infusion with $\text{Alexa647}^{\text{DART}}$, showing ligand capture. DAPI (blue), dTomato-NLS (magenta), $\text{Alexa647}^{\text{DART}}$ (grey). Scale bar, 1000 µm. **(E)** Specificity of AstroHTP in the hippocampus: coronal sections were immunolabeled with antibodies against cell-type-specific markers: SOX9 (astrocytes), NeuN (neurons), and OLIG2 (oligodendrocytes). Scale bar, 100 µm.

bioRxiv preprint doi: https://doi.org/10.64898/2026.10.08.757679; this version posted October 11, 2026. The copyright holder for this preprint (which was not certified by peer review) is the author/funder, who has granted bioRxiv a license to display the preprint in perpetuity. It is made available under a CC-BY 4.0 International license.

Figure S2. Supplement to Figure 2.

Further characterization of astrocyte-specific AMPAR attenuation with YM90K^{DART}.

(A–B) Astrocytes express AMPAR subunits. Summary of published TRAP RNA-seq (*17*) **(A)** and proteomic (*18*) **(B)** datasets reporting glutamate receptor expression in striatal astrocytes. **(C)** YM90K^{DART}-mediated attenuation of AMPAR function at low ambient drug concentrations. The DART tether (blue line) anchors the pharmacological ligand to HTP on target cells, selectively enriching the drug at the cell surface and enabling effective receptor inhibition despite minimal free ligand in the surrounding tissue. Tethered pharmacology is governed by two separable parameters: CC50 (cumulative capture 50%), which defines the exposure (concentration × time, nM·min) required for half-maximal tethered-drug effect on +HTP cells and reflects irreversible HaloTag ligand-driven capture; and AC50 (ambient concentration 50%), which defines the ambient concentration (nM) producing half-maximal reversible drug effect on −HTP cells and is determined by the pharmacological moiety. Consequently, non-HTP-expressing cells (grey outline) experience only low ambient drug levels and remain largely unaffected. Adapted from Shields et al (*11*). **(D–F)** Astrocyte Ca^{2+} response to AMPA application. **(D)** *Upper:* two-photon *ex vivo* Ca^{2+} imaging paradigm in astrocytes. Dorsal striatal (dSTR) astrocytes from adult C57BL/6J mice were co-transduced with AstroHTP and GCaMP6f. Cyclothiazide (CTZ) was applied to block AMPAR desensitization and enhance astrocytic Ca^{2+} responses, and tetrodotoxin (TTX) was applied to block action potential-dependent neuronal activity, isolating the direct astrocytic response to AMPAR activation. *Lower:* representative images of GCaMP6f-expressing astrocytes with ROIs selected for analysis, and corresponding ΔF/F raster plots showing Ca^{2+} activity of each ROI throughout the experiment. **(E)** ΔF/F traces showing astrocytic Ca^{2+} responses to AMPA (100 µM) in the presence of CTZ (100 µM) and TTX (0.5 µM), with or without NBQX. Traces correspond to ACSF control (grey) or NBQX (orange). Shading indicates mean ± SEM. **(F)** NBQX reduced peak Ca^{2+} responses (ACSF 1.08 ± 0.196; NBQX 0.0291 ± 0.00609; unpaired t-test, $P = 0.0063$; N=3 mice, n = 4 slices (ACSF) and 3 slices (NBQX); 34 and 25 ROIs, respectively). **(G)** Astrocytic responses to AMPA are attenuated by YM90K^{DART}. *left:* two-photon *ex vivo* Ca^{2+} imaging paradigm in astrocytes. Dorsal striatal (dSTR) astrocytes from adult C57BL/6J mice were co-transduced with AstroHTP and GCaMP6f. Slices were pre-incubated with DART ligands before application of AMPA with cyclothiazide (CTZ). Tetrodotoxin (TTX) was applied to block action potential-dependent neuronal activity, isolating the direct astrocytic response to AMPAR activation. *right:* representative images of GCaMP6f-expressing astrocytes with ROIs selected for analysis, and corresponding ΔF/F raster plots showing Ca^{2+} activity of each ROI throughout the experiment.

bioRxiv preprint doi: https://doi.org/10.64898/2026.10.08.757679; this version posted October 11, 2026. The copyright holder for this preprint (which was not certified by peer review) is the author/funder, who has granted bioRxiv a license to display the preprint in perpetuity. It is made available under a CC-BY 4.0 International license.

Figure S3 Supplementary for Figure 3.

Turning Behavior and Additional Locomotor Parameters 24 Hours Post DART-Infusion

(A) Cumulative net turns of the mouse, showing both right and left turns over time and their accumulation over the session. Data are mean ± SEM. N = 10 control, 11 YM90K^{DART}-infused mice. **(B)** Fraction of right turns. Control 0.801± 0.0743; YM90K^{DART} 0.628± 0.0718; Mann–Whitney test, P = 0.1517; N = 10 control, 11 YM90K^{DART}-infused mice. **(C)** Turn-diameter histogram (1 cm bins) 24 h after control (grey) or YM90K^{DART} infusion. Left turns are plotted as downward lines and right turns as upward lines. Data are mean ± SEM. N = 10 control, 11 YM90K^{DART}-infused mice. **(D)** Total turns at 24 h post-DART infusion. Mann-Whitney test, P = 0.3494. **(E)** Both groups showed a reduction in distance traveled between 1 h and 24 h post-infusion (two-way ANOVA, main effect of timepoint: F(1,19) = 14.99, P = 0.0010), with no effect of treatment (two-way ANOVA, main effect of treatment: F(1,19) = 0.05, P = 0.8237). **(F)** % Time in center did not differ between control and YM90K^{DART}-treated mice or between time points (two-way ANOVA, main effect of treatment: F(1,19) = 0.6397, P = 0.4337, or effect of time F (1, 19) = 0.1260, P = 0.726. **(G)** Time spent immobile did not differ between groups (two-way ANOVA, main effect of treatment: F(1,19) = 0.08, P = 0.7825) but decreased between 1 h and 24 h in both groups (two-way ANOVA, main effect of timepoint: F(1,19) = 10.72, P = 0.0040).

bioRxiv preprint doi: https://doi.org/10.64898/2026.10.08.757679; this version posted October 11, 2026. The copyright holder for this preprint (which was not certified by peer review) is the author/funder, who has granted bioRxiv a license to display the preprint in perpetuity. It is made available under a CC-BY 4.0 International license.

Figure S4 Supplementary for Figure 3.

Neuronal AMPAR inhibition of iSPNs via YM90K^{DART} is not NMDAR dependent.

(A) Adora2a-Cre mice (iSPNs) were transduced with AAV-CAG-DIO-HTP-2A-dTomato-WPRE or a binding-deficient control, AAV-CAG-DIO-^{dd}HTP-2A-dTomato-WPRE, which cannot capture the HaloTag ligand and therefore leaves YM90K^{DART} untethered. AMPA-R mediated eEPSC were recorded from striatal slices in the ACSF solution containing picrotoxin (50 µM) and APV (10 µM), and bath-applied with 300 nM YM90K^{DART}/30 nM Alexa647^{DART} after a 7.5-minute baseline. Recordings were obtained from neurons expressing HTP or ^{dd}HTP (control). **(B)** Representative image of iSPNs expressing HTP (red), capturing Alexa647DART (grey). Scale bar, 10 µm. **(C)** Time course of normalized eEPSC amplitude showing a marked reduction following YM90K^{DART} treatment in HTP-expressing cells (red) compared with ^{dd}HTP-expressing cells (grey). Data are mean ± SEM. ^{dd}HTP condition: 8 cells from 4 mice, $^{+}$HTP condition: 7 cells from 3 mice. **(D)** Representative eEPSC traces at baseline (black) and after YM90K^{DART} application (red), shown for both ^{dd}HTP and $^{+}$HTP cells. **(E)** eEPSC amplitude was significantly reduced following YM90K^{DART} treatment (control 87.4 ± 4.45%; YM90K^{DART} 30.2 ± 8.37%; Mann–Whitney test, P = 0.0003; 17.5–22.5 min). ^{dd}HTP condition: 8 cells from 4 mice, $^{+}$HTP condition: 7 cells from 3 mice.

bioRxiv preprint doi: https://doi.org/10.64898/2026.10.08.757679; this version posted October 11, 2026. The copyright holder for this preprint (which was not certified by peer review) is the author/funder, who has granted bioRxiv a license to display the preprint in perpetuity. It is made available under a CC-BY 4.0 International license.

Figure S5 Supplementary for Figure 3.

Astrocytic AMPAR inhibition of SPNs does not alter basal synaptic transmission.

(A) Experimental design for mEPSC recordings. **(B)** Representative recording configuration showing a GFP-positive dSPN (green) patched adjacent to an NLS-dTomato-positive astrocyte (red). Scale bar, 10 µm. **(C)** Distance between patched dSPNs and neighboring astrocytes did not differ between groups (control 9.67 ± 1.25 µm; $YM90K^{DART}$ 8.17 ± 0.852 µm; Mann–Whitney test, P = 0.4463; control n = 15 cells from 4 mice; $YM90K^{DART}$ n = 16 cells from 4 mice. **(D)** Representative mEPSC traces recorded from dSPNs in control (grey) and $YM90K^{DART}$ treated (blue) conditions. **(E)** mEPSC amplitude in dSPNs was unchanged (control 10.2 ± 0.345 pA; $YM90K^{DART}$ 9.70 ± 0.288 pA; Mann–Whitney test, P = 0.4008; control n = 15 cells from 4 mice; $YM90K^{DART}$ n = 16 cells from 4 mice). **(F)** mEPSC frequency in dSPNs was unchanged (control 0.897 ± 0.129 Hz; $YM90K^{DART}$ 1.31 ± 0.296 Hz; Mann–Whitney test, P = 0.8609; control n = 15 cells from 4 mice; $YM90K^{DART}$ n = 16 cells from 4 mice). **(G)** Representative recording configuration showing a GFP-positive iSPN (green) patched adjacent to an NLS-dTomato-positive astrocyte (red). Scale bar, 10 µm **(H)** Distance between patched iSPNs and neighboring astrocytes did not differ between groups (control 8.29 ± 0.987 µm; $YM90K^{DART}$ 8.23 ± 1.07 µm; Mann–Whitney test, P = 0.9668; control n = 20 cells from 4 mice; $YM90K^{DART}$ n = 19 cells from 4 mice). **(I)** Representative mEPSC traces recorded from iSPNs in control (grey) and $YM90K^{DART}$ treated (blue) conditions. **(J)** mEPSC amplitude in iSPNs was unchanged (control 9.12 ± 0.258 pA; $YM90K^{DART}$ 9.65

± 0.244 pA; Mann–Whitney test, P = 0.1841; control n = 20 cells from 4 mice; $YM90K^{DART}$ n = 19 cells from 4 mice). **(K)** mEPSC frequency in iSPNs was unchanged (control 0.399 ± 0.0413 Hz; $YM90K^{DART}$ 0.427 ± 0.0435 Hz; Mann–Whitney test, P = 0.3803; control n = 20 cells from 4 mice; $YM90K^{DART}$ n = 19 cells from 4 mice).

bioRxiv preprint doi: https://doi.org/10.64898/2026.10.08.757679; this version posted October 11, 2026. The copyright holder for this preprint (which was not certified by peer review) is the author/funder, who has granted bioRxiv a license to display the preprint in perpetuity. It is made available under a CC-BY 4.0 International license.

Figure S6 Supplementary for Figures 4 and 5.

Astrocyte AMPAR Blockade Reduces eEPSCs via NMDAR-Dependent, D-Serine-Sensitive Mechanism.

(A) Comparison of the change in eEPSC amplitude after 10 min of stimulation (ΔeEPSC, % of baseline): continuous −21.7 ± 8.62%; paused −37.2 ± 13.1% (Mann–Whitney test, P = 0.4234; continuous n = 9 cells from 5 mice, paused n = 8 cells from 4 mice). **(B)** Time course of the first eEPSC amplitude in the PPR experiment, normalized to baseline. YM90K^{DART} treatment reduced eEPSC amplitude.). Both measures: n = 9 cells from 5 mice. **(C)** Time course and quantification of PPR, showing no change following YM90K^{DART} treatment. **(D)** Paired comparison of eEPSC amplitude at baseline and after 10 min of YM90K^{DART} application, 64.4% ± 8.16 % (Wilcoxon test, P = 0.0034), n=12 cells, 10 mice. **(E)** Paired comparison of eEPSC amplitude at baseline and 10–20 min post-infusion of YM90K^{DART} and MK-801 , 80.3%± 7.62% (Wilcoxon test, P = 0.0134), n=13 cells, 9 mice. **(F)** Paired comparison of eEPSC amplitude at baseline and 10–20 min post-infusion of YM90K^{DART} and MK-801+D-serine, 108%± 5.58% (Wilcoxon test, P = 0.2061), n=11 cells, 9 mice. **(G)** Paired comparison of eEPSC amplitude at baseline and 10–20 min post-infusion of vehicle, 105%± 9.96% (Wilcoxon test, P = 0.6377), n=11cells, 7 mice. **(H)** Paired comparison of eEPSC amplitude at baseline and 10–20 min post-infusion of DAAO, 72.7%± 7.40% (Wilcoxon test, P = 0.0040), n=14 cells, 8 mice.

bioRxiv preprint doi: https://doi.org/10.64898/2026.10.08.757679; this version posted October 11, 2026. The copyright holder for this preprint (which was not certified by peer review) is the author/funder, who has granted bioRxiv a license to display the preprint in perpetuity. It is made available under a CC-BY 4.0 International license.

Supplementary Videos: 2P imaging of astrocyte calcium transients in response to AMPA bath application.

Supp Video 1. Representative time-lapse video showing astrocyte response to application of 100uM AMPA+100uM CTZ

Supp Video 2. Representative time-lapse video showing astrocyte lack of response to application of 100uM AMPA+100uM CTZ pre-incubated in 30uM NBQX

Supp Video 3. Representative time-lapse video showing astrocyte response to application of 100uM A MPA+100uM CTZ pre-incubated in 1uM Blank ^{DART}.

Supp Video 4. Representative time-lapse video showing astrocyte lack of response to application of 100uM A MPA+100uM CTZ pre-incubated in 1uM YM90K^{DART}.

Supplementary Videos: 2P imaging of astrocyte spoteneuos calcium transients

Supp Video 5. Representative time-lapse video showing AquA2 analysis of spoteneuos calciuym events in slices pre-incubated in 1uM Blank ^{DART}.

Supp Video 6. Representative time-lapse video showing AquA2 analysis of spoteneuos calcium events in slices pre-incubated in 1uM YM90K^{DART}.

Supp Video 7. Representative time-lapse video showing DANNCE tracking of mice in open field.
