# Supplementary material for: An astrocytic AMPAR-dependent parity signal preserves synaptic strength
Source: bioRxiv. 2026 Oct 11:2026.10.08.757679. Preprint. [Version 1] doi: 10.64898/2026.10.08.757679 (PMC13692175; doi:10.64898/2026.10.08.757679)
Supplement: Supplementary file 2 [file media-1.pdf]

figure S1

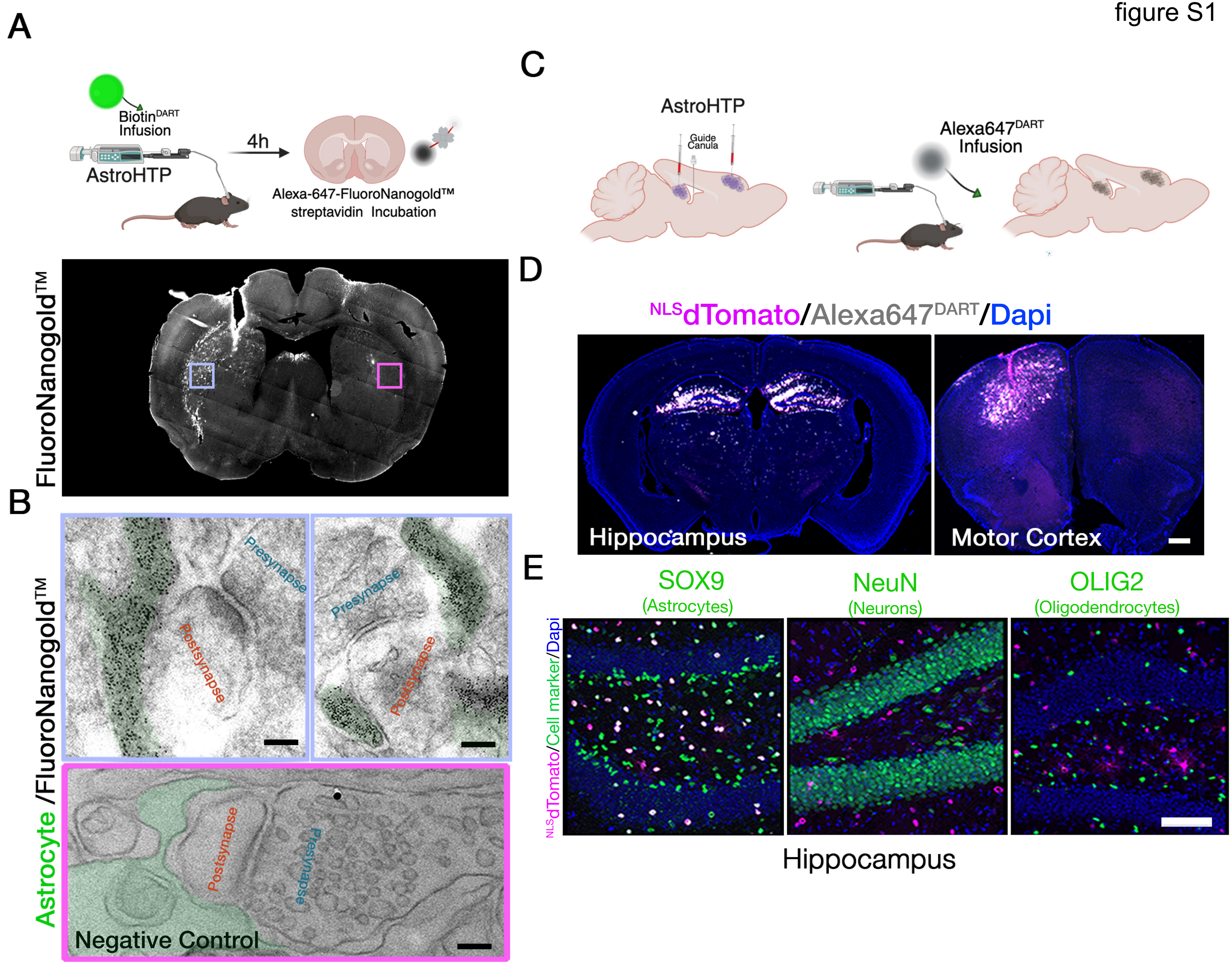

A
Biotin^{DART} Infusion
AstroHTP
4h
Alexa-647-FluoroNanogold™ streptavidin Incubation
FluoroNanogold™
B
Astrocyte /FluoroNanogold™
Presynapse
Postsynapse
Negative Control
C
AstroHTP
Guide Canula
Alexa647^{DART} Infusion
D
^{NLS}dTomato/Alexa647^{DART}/Dapi
Hippocampus
Motor Cortex
E
SOX9 (Astrocytes)
NeuN (Neurons)
OLIG2 (Oligodendrocytes)
^{NLS}dTomato/Cell marker/Dapi
Hippocampus

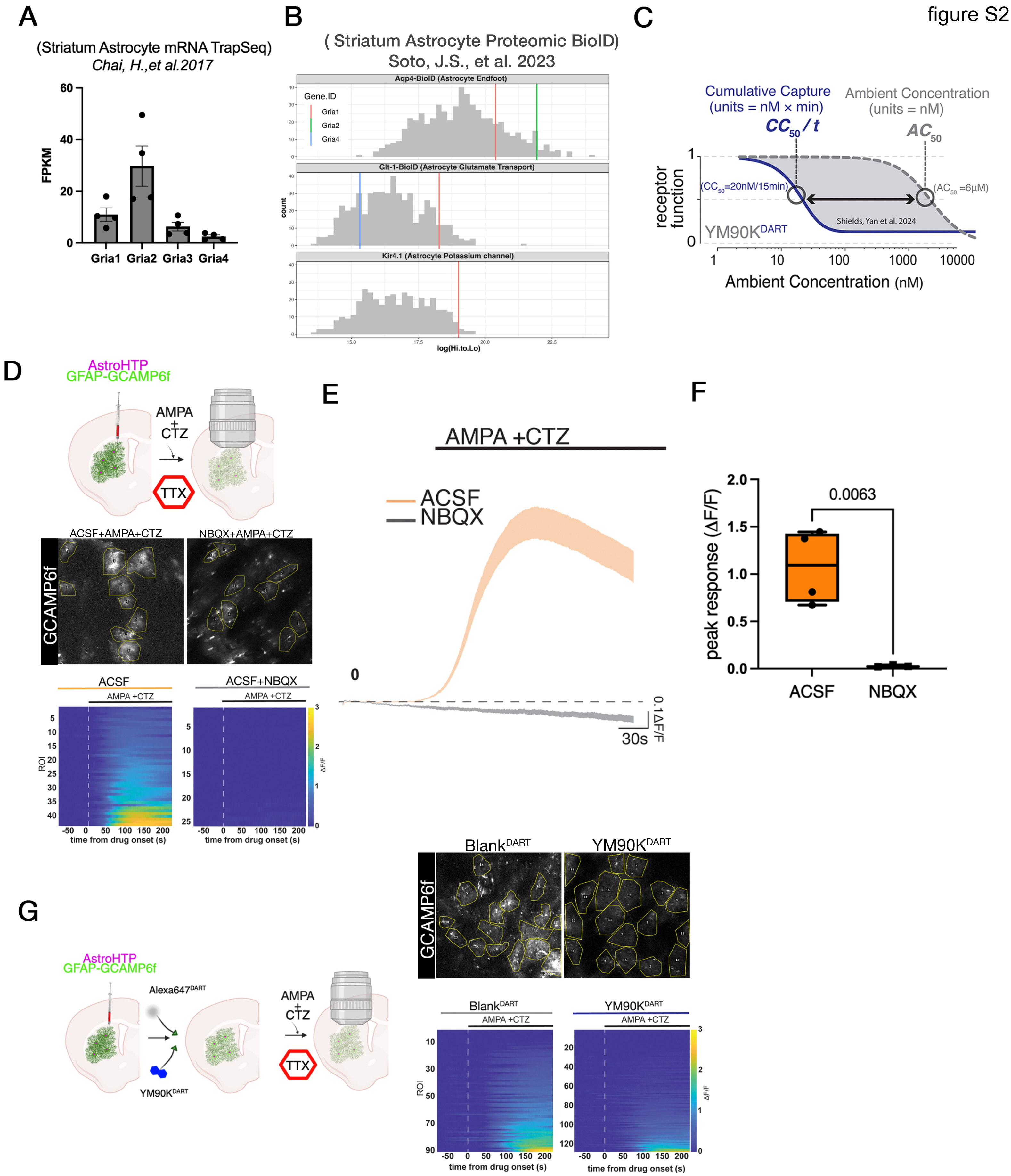
A
(Striatum Astrocyte mRNA TrapSeq)
Chai, H.,et al.2017
FPKM
Gria1 Gria2 Gria3 Gria4
B
(Striatum Astrocyte Proteomic BioID)
Soto, J.S., et al. 2023
Aqp4-BioID (Astrocyte Endfoot)
Gene.ID
Gria1
Gria2
Gria4
Glt-1-BioID (Astrocyte Glutamate Transport)
Kir4.1 (Astrocyte Potassium channel)
count
log(Hi.to.Lo)
C
Cumulative Capture
(units = nM × min)
CC50 / t
Ambient Concentration
(units = nM)
AC50
receptor function
(CC50=20nM/15min)
(AC50 =6μM)
Shields, Yan et al. 2024
YM90KDART
Ambient Concentration (nM)
D
AstroHTP
GFAP-GCAMP6f
AMPA + CTZ
TTX
ACSF+AMPA+CTZ
NBQX+AMPA+CTZ
GCAMP6f
ACSF
ACSF+NBQX
AMPA +CTZ
ROI
ΔF/F
time from drug onset (s)
E
AMPA +CTZ
ACSF
NBQX
0.1ΔF/F
30s
F
peak response (ΔF/F)
0.0063
ACSF
NBQX
G
AstroHTP
GFAP-GCAMP6f
Alexa647DART
YM90KDART
AMPA + CTZ
TTX
BlankDART
YM90KDART
GCAMP6f
AMPA +CTZ
ROI
ΔF/F
time from drug onset (s)

figure S3

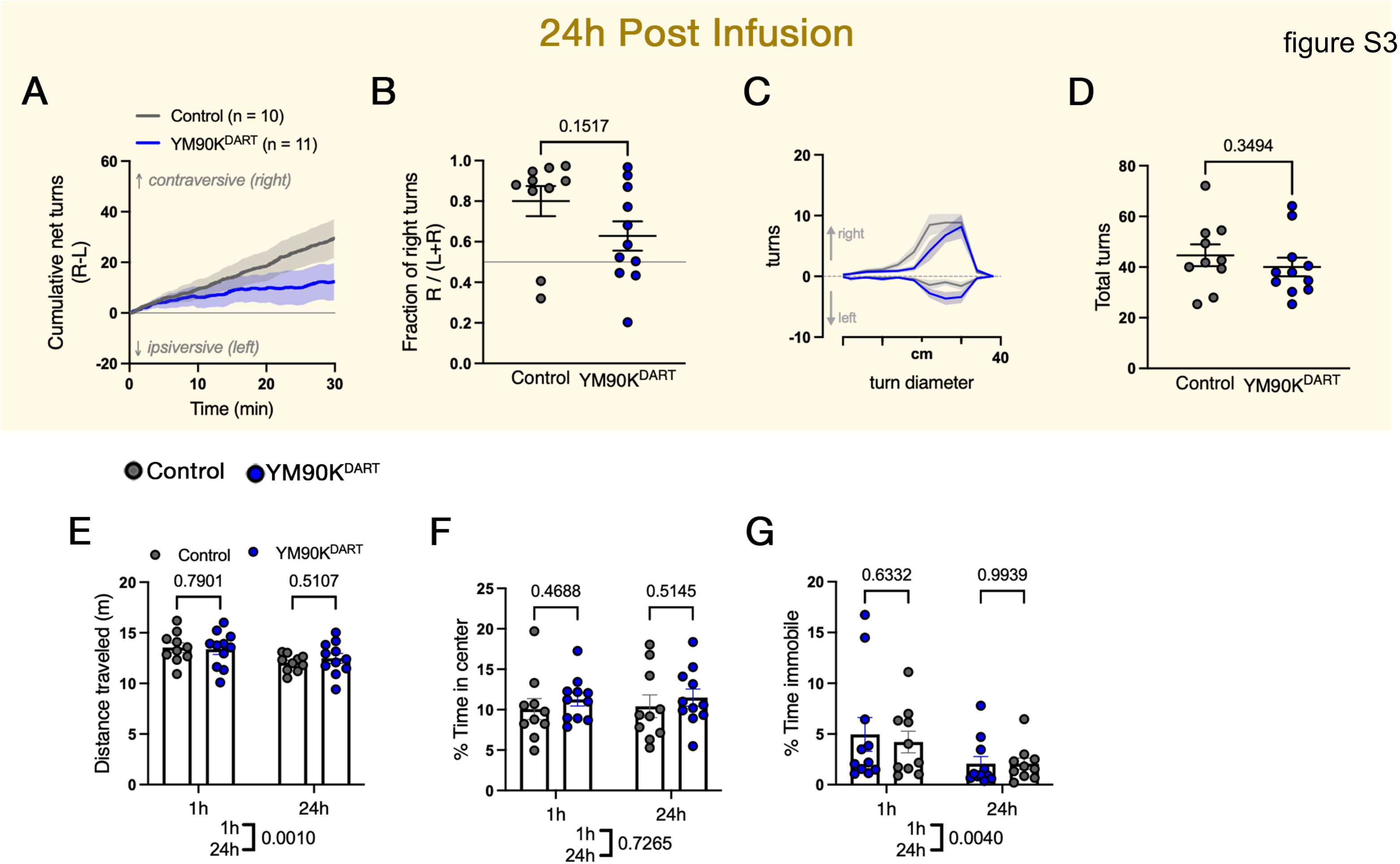
24h Post Infusion
A
Control (n = 10)
YM90K^DART (n = 11)
↑ contraversive (right)
↓ ipsiversive (left)
Cumulative net turns (R-L)
Time (min)
B
0.1517
Fraction of right turns R / (L+R)
Control
YM90K^DART
C
right
left
turns
cm
turn diameter
D
0.3494
Total turns
Control
YM90K^DART
Control
YM90K^DART
E
Control
YM90K^DART
0.7901
0.5107
Distance traveled (m)
1h
24h
1h
24h
0.0010
F
0.4688
0.5145
% Time in center
1h
24h
1h
24h
0.7265
G
0.6332
0.9939
% Time immobile
1h
24h
1h
24h
0.0040

figure S4

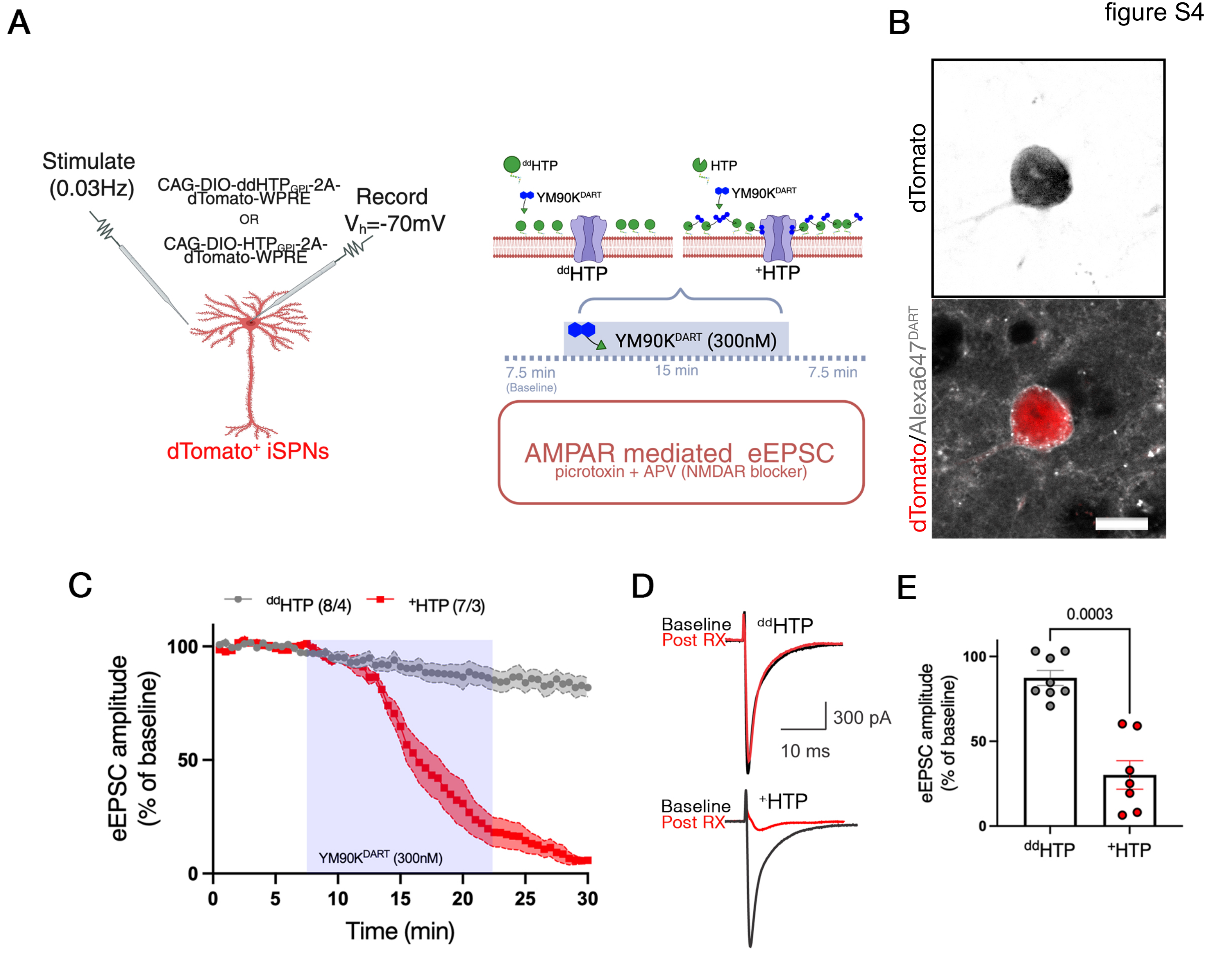
A
Stimulate (0.03Hz)
CAG-DIO-ddHTP$_{GPI}$-2A-dTomato-WPRE
OR
CAG-DIO-HTP$_{GPI}$-2A-dTomato-WPRE
Record
V_h=-70mV
dTomato+ iSPNs
ddHTP
YM90K^{DART}
HTP
YM90K^{DART}
ddHTP
+HTP
YM90K^{DART} (300nM)
7.5 min
(Baseline)
15 min
7.5 min
AMPAR mediated eEPSC
picrotoxin + APV (NMDAR blocker)
B
dTomato
dTomato/Alexa647^{DART}
C
ddHTP (8/4)
+HTP (7/3)
eEPSC amplitude (% of baseline)
100
50
0
0
5
10
15
20
25
30
Time (min)
YM90K^{DART} (300nM)
D
Baseline
Post RX
ddHTP
300 pA
10 ms
Baseline
Post RX
+HTP
E
0.0003
eEPSC amplitude (% of baseline)
100
50
0
ddHTP
+HTP

figure S5

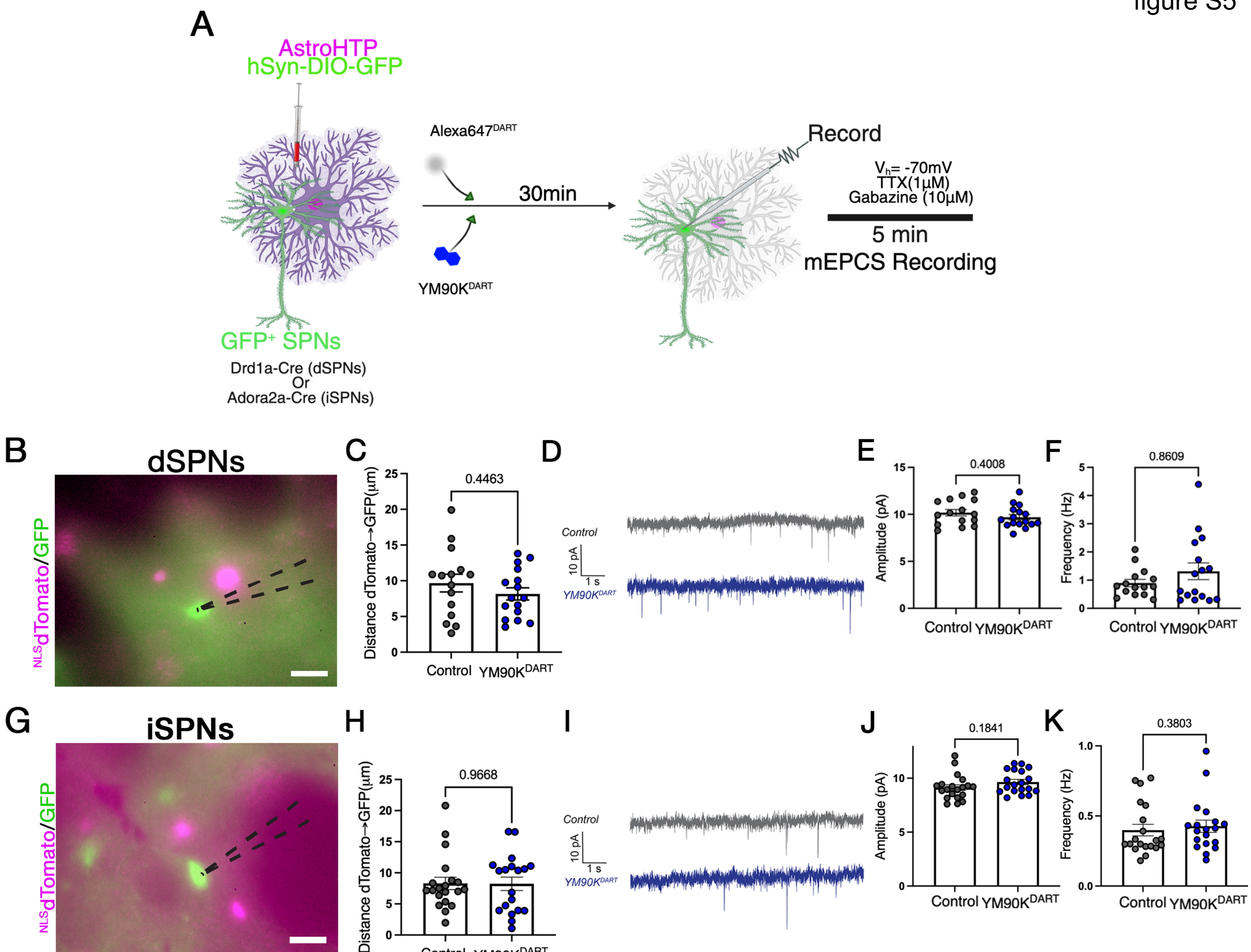
A
AstroHTP
hSyn-DIO-GFP
GFP+ SPNs
Drd1a-Cre (dSPNs)
Or
Adora2a-Cre (iSPNs)
Alexa647DART
YM90KDART
30min
Record
Vh= -70mV
TTX(1µM)
Gabazine (10µM)
5 min
mEPCS Recording
B
dSPNs
NLSdTomato/GFP
C
Distance dTomato→GFP(µm)
0.4463
Control
YM90KDART
D
Control
YM90KDART
10 pA
1 s
E
Amplitude (pA)
0.4008
F
Frequency (Hz)
0.8609
G
iSPNs
H
0.9668
YM90KDART.
I
J
0.1841
K
0.3803

figure S6

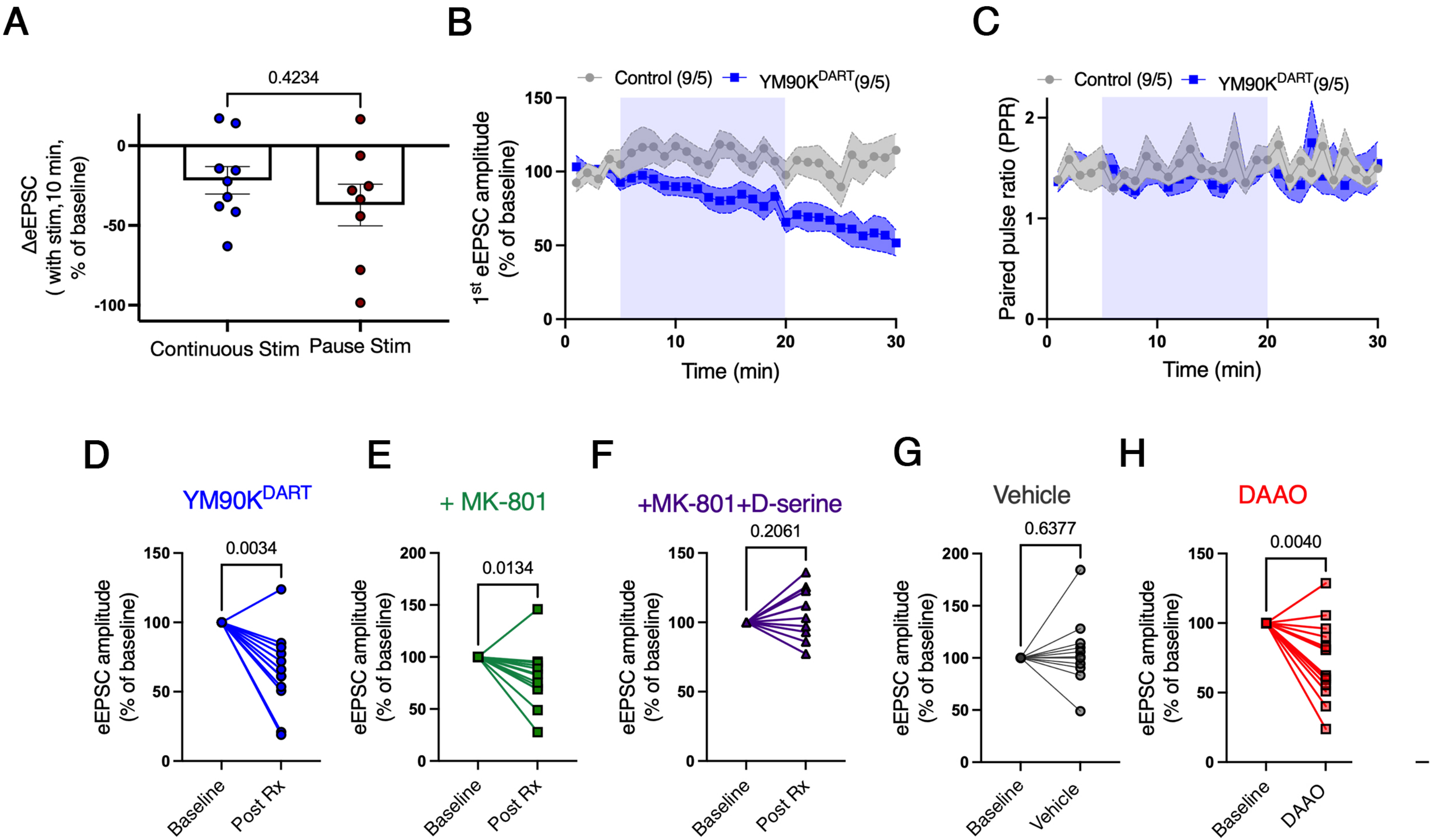
A
0.4234
ΔeEPSC
(with stim,10 min,
% of baseline)
0
-50
-100
Continuous Stim
Pause Stim
B
Control (9/5)
YM90K^DART(9/5)
150
100
50
0
1st eEPSC amplitude
(% of baseline)
0
10
20
30
Time (min)
C
Control (9/5)
YM90K^DART(9/5)
Paired pulse ratio (PPR)
2
1
0
0
10
20
30
Time (min)
D
YM90K^DART
0.0034
eEPSC amplitude
(% of baseline)
150
100
50
0
Baseline
Post Rx
E
+ MK-801
0.0134
200
150
100
50
0
Baseline
Post Rx
F
+MK-801+D-serine
0.2061
150
100
50
0
Baseline
Post Rx
G
Vehicle
0.6377
200
150
100
50
0
Baseline
Vehicle
H
DAAO
0.0040
150
100
50
0
Baseline
DAAO
